# A Gravitational-like Relationship of Dispersion Interactions is Exhibited by 40 Pairs of Molecules and Noble Gas Atoms

**DOI:** 10.1021/jacs.4c11211

**Published:** 2024-10-31

**Authors:** David Danovich, Alexandre Tkatchenko, Santiago Alvarez, Sason Shaik

**Affiliations:** † Institute of Chemistry, The Hebrew University of Jerusalem, Jerusalem 9190401, Israel; ‡ Department of Physics and Materials Science, University of Luxembourg, L-1511 Luxembourg City, Luxembourg; § Inorganic Chemistry Department, Facultat de Química, Universitat de Barcelona, Martí i Franquès 1-11, 08028 Barcelona, Spain

## Abstract

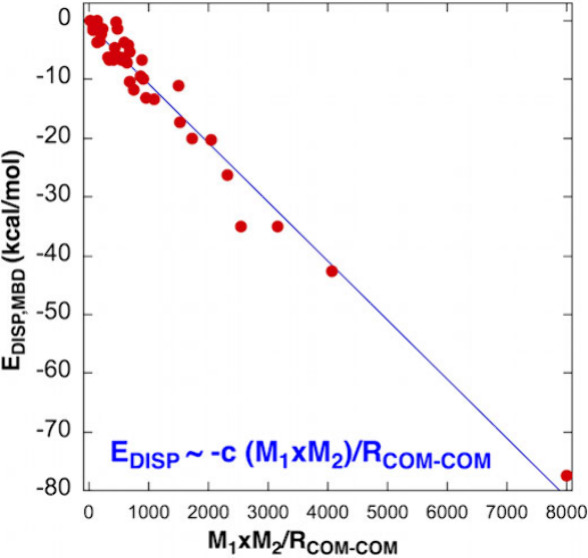

We present computational results of many-body dispersion (MBD) interactions for 40 pairs of molecular and atomic species: hydrocarbons, silanes, corresponding fluorinated derivatives, pairs which have multiple H---H contacts between the molecules, as well as pairs having π–π interactions, and pairs of noble gases. The calculations reveal that the MBD stabilization energy (*E*_DISP,MBD_) obeys a global relationship, which is *gravitational-like*. It is proportional to the product of the masses of the two molecules (*M*_1_*M*_2_) and inversely proportional to the corresponding distances between the molecular centers-of-mass (*R*_COM-COM_) or the H---H distances of the atoms mediating the interactions of the two molecules (*R*_H–H_). This relationship reflects the interactions of instantaneous dipoles, which are formed by the ensemble of bonds/atoms in the interacting molecules. Using the D4-corrected dispersion energy (*E*_DISP,D4_), which accounts for three-body interactions, we find that the *E*_DISP,MBD_ and *E*_DISP,D4_ data sets are strongly correlated. Based on valence-bond modeling, the dispersion interactions occur primarily due to the increased contributions of the oscillating-ionic VB structures which maintain favorable electrostatic interactions; the [Sub—C^+^:H^–+^H:C^–^—Sub] and [Sub—C:^–+^H ^–^H:C^+^—Sub] structures; Sub symbolizes general residues. This augmented contribution is complemented by simultaneously diminished-weights of the destabilizing pair of structures, [Sub—C^+^:H^––^H:C^+^—Sub] and [Sub—:C^–^ H^++^H:C^–^—Sub]. The local charges are propagated to the entire ensemble of bonds/atoms by partially charging the Sub residues, thus bringing about the “gravitational-like” dependence of dispersion.

## 1 Introduction

Unlike hydrogen bonds, which involve localized interactions,^1,2^ the van der Waals (vdW) *dispersion-interactions are cumulative*, and may involve all the atoms/bonds in the interacting molecules. As such, dispersion is a major design factor; it is a force of nature which stabilizes condensed matter in chemistry and biology.

Indeed, dispersion interactions have attracted considerable attention in the past two decades or so.^3−25^ Essentially, the *global dispersion interaction* in molecular systems is a many-body electronic-effect that arises from electron density fluctuations in the ensemble of atoms/bonds.^5,6^

**Figure 1:**
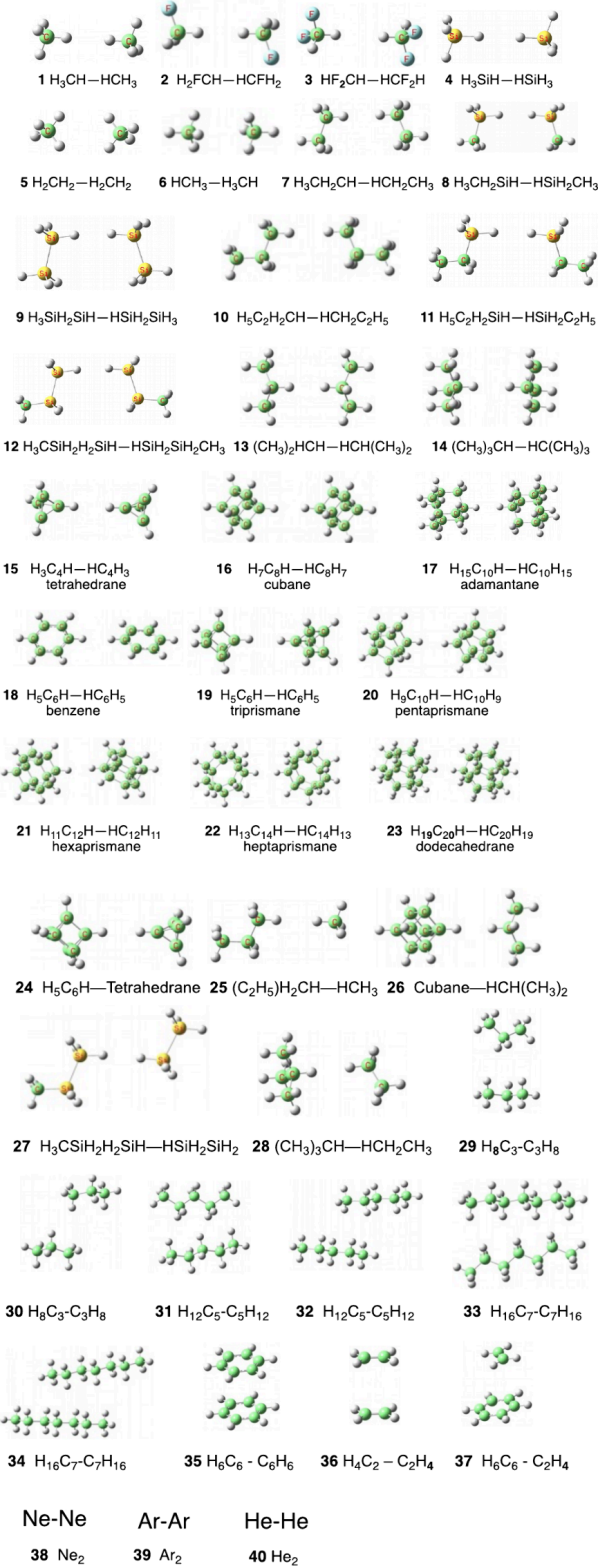
Homo- and heterodimers **1**–**40**, which are studied using PBE0/cc-pVTZ with MBD and D4 dispersion corrections (C is green, F is bluish, and Si is yellow). The geometries are fully optimized at the PBE0-MBD/cc-pVTZ and PBE0-D4/cc-pVTZ levels of theory.

**Figure 2:**
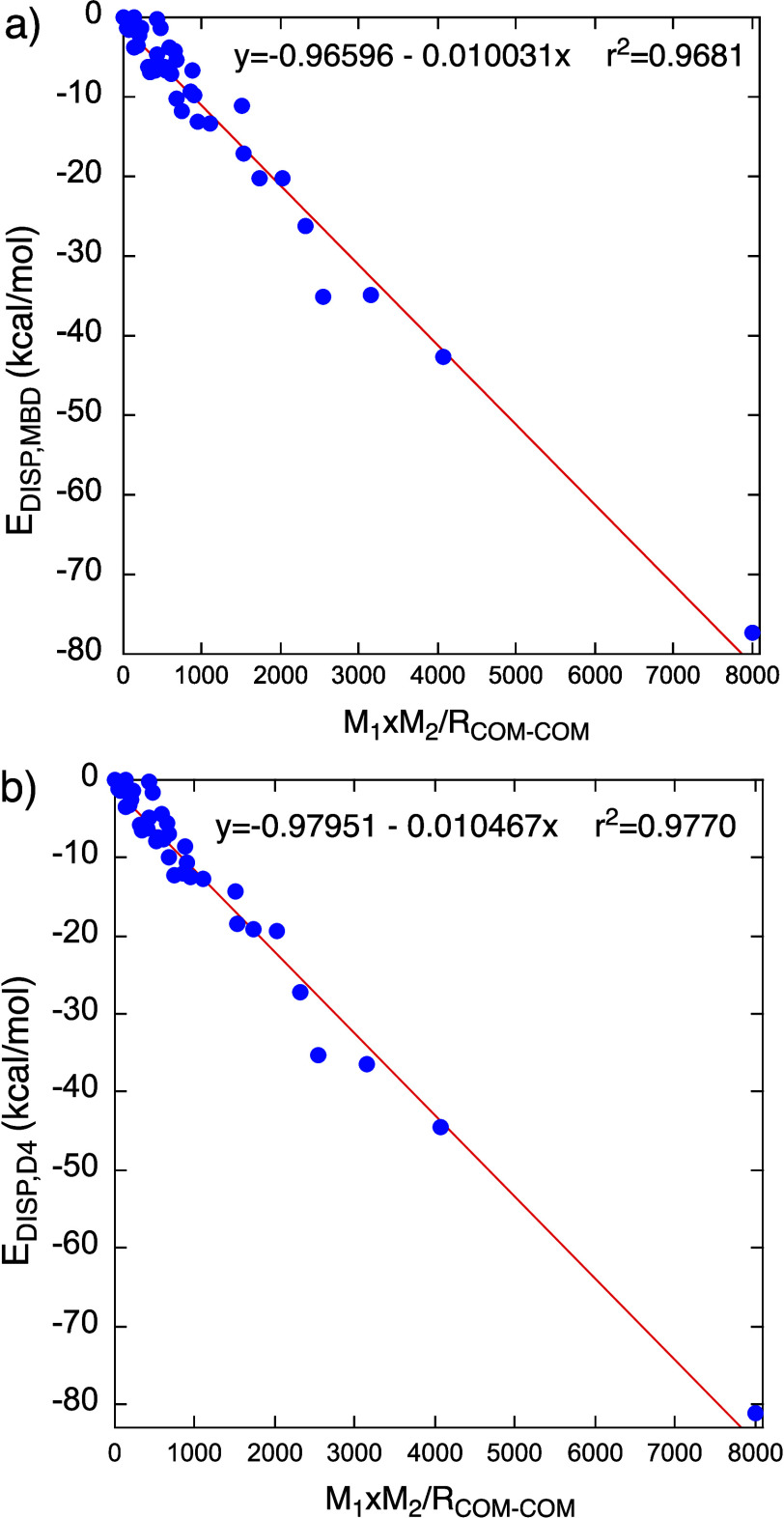
Dependence of the dispersion interaction energy (*E*_DISP_) on the gravitational-like expression *M*_1_*M*_2_/*R*_COM-COM_, where *M*_1_ and *M*_2_ are the molecular masses of the molecules/atoms in the dimer and *R*_COM-COM_ is the distance between the respective centers of these masses: (a) Using the many-body dispersion (MBD) method (*E*_DISP, MBD_ (kcal/mol)). (b) Using the D4 dispersion method (*E*_DISP,D4_ (kcal/mol)).

**Figure 3:**
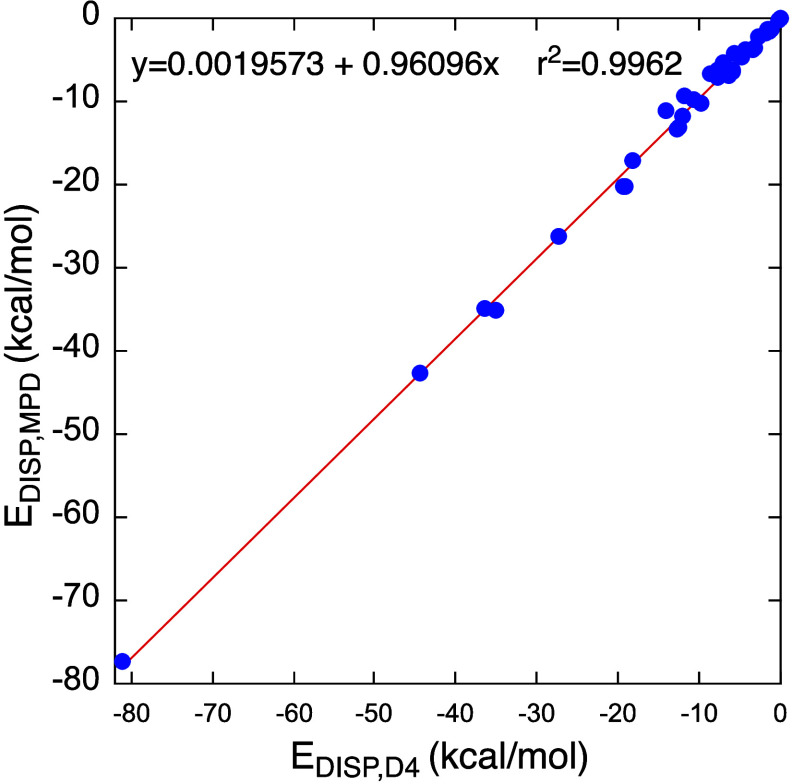
Correlation between dispersion energies calculated by the MBD (*E*_DISP,MBD_) vs the D4 (*E*_DISP,D4_) methods.

The presently available many-body dispersion (MBD) software,^6^ and Grimme’s D4 method^12^ can be coupled to electronic-calculation codes to calculate dispersion energies (*E*_DISP_). Doing so, we show here that the dispersion interactions between homodimers and heterodimers of alkanes and silanes (see Figure 1), including fluorinated derivatives, rings, noble atom-dimers, π–π interacting dimers, 3D objects etc., obey a gravitational-like law. Thus, *E*_DISP_*is shown to be proportional to the product of the two molecular masses (M*_1_*M*_2_*), of the interacting molecules/atoms, and inversely proportional to their distances* (see Figures 2 and 3). This expression emerges from the contribution of dispersion interactions by all the bonds/atoms in the studied molecules/atoms. Understanding the nature of *E*_DISP_ is clearly essential.

## 2 Methods

We use MBD^6^- and D4^12^-coupled to density functional (DFT) calculations, PBE0/cc-pVTZ,^26−28^ as implemented in the QCHEM-6.02 package.^29^ The method is applied to 40 pairs of molecules/atoms, and demonstrates that the global dispersion energy obeys a gravitational-like law. This relationship indicates that the vdW dispersion-interaction reflects the entire ensemble of bonds/atoms in the studied dimers.^5,10,17^

## 3 Results and Discussions

### 3.1 Dispersion Interactions in Substituted Hydrocarbons and Higher Row Analogues

In hydrocarbons, the homopolar dihydrogen CH---HC interactions lack the electrostatic component that characterize the heteropolar dihydrogen bonds defined by Crabtree et al.^30^ Since the molecular-pairs studied here are all of the homopolar type, we will refer to CH---HC simply as “contacts” or “interactions”. These contacts serve as “sticky fingers” in condensed phases and in molecular dimers.^13−15^ Thus, for example, unlike small hydrocarbons which are gaseous molecules at room temperature (e.g., CH_4_), large hydrocarbons like dodecahedrane and other polyhedranes form condensed phases and solids^13,14^ with melting points that reach as high as 723 K.

The recent review of Rumel and Schreiner^17^ provides compelling evidence for the stabilizing role of dispersion and the molecular drive for creating congested geometries. Furthermore, Chen and co-workers^22^ showed that the drive for adaptation of congested and dispersion-stabilized molecules, persists in solvents, albeit getting somewhat attenuated. Similarly, substituted ethane molecules, which possess very long C–C bonds, that are virtually semibroken, e.g., as in hexa-(3,5-di-t-butylphenyl)-ethane,^16−19^ are nevertheless held together, due to the dispersion interactions of the large *t*-butyl substituents, which stabilize the molecule by ca. 40 kcal/mol.^17−19^ Moreover, graphane layers are stabilized via σ–σ and π–π dispersions (and orbital interactions) by as much as 150 kcal/mol per a pair of layers^20,21^

High level ab initio calculations are required to reproduce such weak CH---HC dispersion interactions.^14,23−25^ However, in large molecules, such as dodecahedrane, one has to use DFT calculations (herein, PBE0^26^). DFT calculations generally (though not always^31^) require dispersion corrections, as an add-on to the DFT energies; hence, corresponding to DFT-D or DFT+vdW calculations.^9−12,17^

There are a few general dispersion-inclusive methods; one which was pioneered by Grimme and his co-workers,^9−12^ is the semiempirical D3 dispersion correction,^11^ and the more recent D4 correction^12^ that accounts for three-body interactions. The second type, which was formulated by Tkatchenko et al.,^3−8,32−38^ involves MBD effects, which in principle account for the contributions *of all the atoms/bonds* in the molecular ensemble. The third type was developed by Neese et al.,^23−25^ as part of an ab initio energy decomposition analysis, *which evaluates the intermolecular dispersion interactions*. As our interest is in the global dispersion energy, we use here the first two types.

Figure 1 displays the 40 dimers which are studied herein, and which involve H---H intermolecular contacts between hydrocarbons and silanes of various forms and shapes, including chains, substituted chains, rings, cages, etc. Note that **25**-**28** are heterodimers. In addition, some of the dimers (**5**, **6**, **29**-**34**) involve multiple H---H contacts. To broaden the interaction types, we added dimers **35**-**37**, which involve π–π interactions, and noble-gas pairs **38**-**40**. All the structures of the dimers were fully optimized without any constraints and are minima of the corresponding dimers. For most dimers, we have located the minima that possesses H---H contacts, following X-ray structures of similar systems.^14^ For dimers **1**, **5**, **6** we have checked that the dimer **6** with three H---H contacts is a global minimum, while **1** and **5** are local minima of methane dimer. Furthermore, we have verified that systems **1**, **5**, **6**, and **29**-**34** are on the same correlation line *regardless of the geometry*. This is so, because when the geometry changes, the *R*_COM-COM_ changes accordingly.

As shown below, this *variegated ensemble lies on a single correlation line*, when *E*_DISP_ is plotted against M_1_M_2_/*R* where *R* is the distance between the chains; *R* can be used as the distance across the nearest H---H contacts (*R*_H–H_) of the dimers, or preferably the *R*_COM-COM_ distance between the centers of mass (COM) of the monomers in the respective dimers in Figure 1.

The COM of each monomer in the corresponding dimer was calculated using fully optimized geometry of the dimer. *R*_COM-COM_ was calculated at the equilibrium distance between the monomers. Computational data, which affirm the superiority of this particular “gravitational” relationship, are relegated to the Supporting Information (SI) document.

These 40 dimers are subjected to calculations of the dispersion energies (*E*_DISP_) using the MBD (*E*_DISP,MBD_)^5,6^ and D4 (*E*_DISP,D4_)^12^ methods. The respective values are plotted in Figures 2a and 2b correspondingly vs *M*_1_*M*_2_/*R*_COM-COM_, where *R*_COM_-_COM_ can serve as a general unbiased distance parameter. *M*_1_ and *M*_2_ are the masses of each monomer in the corresponding dimer. Finally, Figure 3 plots the dispersion energies calculated by the MBD method vs the D4 values.

It is apparent from Figure 3 that the *E*_DISP,MBD_ and *E*_DISP,D4_ values correlate strongly with one another. Furthermore, the absolute magnitudes of the two dispersion quantities are close to within 1.5 kcal/mol (except of dodecahedrane dimer (**23**) in which the discrepancy is around 3.7 kcal/mol; see Table S2 in the SI). This includes all the dimer varieties **5**, **6**, and **29**-**34**, which involve multiple H---H contacts. Clearly, MBD and D4 yield equivalent dispersion energies for the systems studied in this work. We remark that for larger molecules and supramolecular systems with more than 100 atoms, the differences between MBD and D4 may amount to more than 10 kcal/mol.^36,37^

Thus, the dispersion interaction here is moderately long-range, and it includes all the atoms of the monomers, by induction of charges throughout the interacting molecules.

Using the expressions for the straight-lines in Figure 2, we approximate the dispersion interactions at the PBE0-MBD/cc-pVTZ and PBE0-D4/cc-pVTZ levels, by use of eq 1 (*M*_1_ and *M*_2_ in amu (atomic mass unit), *R*_COM-COM_ in Å). This expression correlates well (*r*^2^ = 0.974) with the data in Figure 2
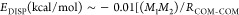
1To avoid bias, we also tried to correlate the *E*_DISP_ data with *the sum of the two masses* (*M*_1_+*M*_2_), as well as, *with the sum of masses divided by the R*_*COM-COM*_*distances*. However, the qualities of these correlations are inferior to those which are obtained, respectively, with the product of the masses (*M*_1_*M*_2_) as well as with the corresponding gravitational relationship (*M*_1_*M*_2_)/*R* (see SI for the respective data, Figures S6a-S6c). Furthermore, we also fitted the dispersion energy to *M*_1_*M*_2_/*R*_COM-COM_^n^ wherein *n* was freely optimized. We obtained that the best correlation fitting with *r*^2^ = 0.97515 is with *n* = 1.2325, quite close to 1 (and so are the respective r^2^ values).

### 3.2 Origins of the Gravitational Relation in eq 1

*The gravitational expression in*eq 1*has a profound message, namely, that molecules/noble-gas atoms, whichever they may be, interact with one another in proportion to their molecular masses*. This is so because the dispersion interaction involves charge-switching in the entire ensemble of atoms/bonds in the two molecules. This mass-dependent dispersion-energy expression (eq 1) can also be derived directly from theory. Thus, based on the seminal London dispersion formula between two atoms or molecules, 1 and 2
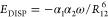
2where α_1_and α_2_ are the static polarizabilities of the interacting moieties, ω is an effective oscillation frequency of the system, and *R*_12_ is the distance between the systems. The frequency ω can be written as *v*/*R*_12_, where *v* is an effective propagation speed of the vdW interaction (*v* is equal to the speed of light for infinite separation between the moieties). The polarizabilities of molecules, α_1,2,_ are in turn proportional to the volume occupied by the molecules, i.e., eq 3:^32^
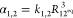
3where  is the equilibrium separation distance. Substituting these expressions into the London formula at the equilibrium distance yields
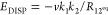
4The appearance of the masses in eq 1 may be rationalized by the term *k*_1_*k*_2_ (eq 4), which scales with the sizes of the interacting molecules. Hence, one can rationalize the emergence of the gravitational-like potential (eq 1) for the dispersion interaction between two atoms/molecules at equilibrium separation.

### 3.3 Understanding the Nature of the Dispersion Interactions

To get further insight into this intriguing correlation, we use valence bond (VB) theory, and focus on the breathing orbital VB (BOVB) approach, which includes both static and dynamic electron-correlations. As such, BOVB gives rise to different orbitals for different VB structures, and hence it involves instantaneous adaptation of the state-wave function to the electron density fluctuations inherent in the ensemble of VB structures.^39−41^ The BOVB method was demonstrated before^14^ to involve dispersion corrections in hydrocarbons and to offer pictorial physical insight into the origins of these interactions. Therefore, using the VB results, we can rationalize the “gravitational-like relationship”, which we found in the DFT-MBD and DFT-D4 data, which essentially reflect *the global interaction of the instantaneous oscillating dipoles that are mediated by the entire ensemble of bonds/atoms within the interacting molecules*, and stabilize thereby the dimers.

**Figure 4:**
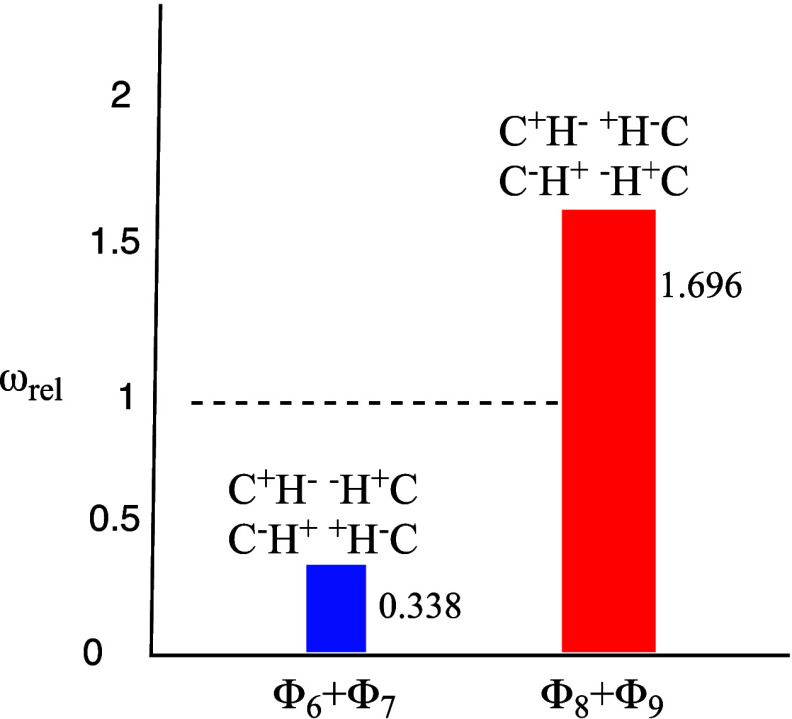
BOVB computed relative weights, ω_rel_ (with respect to dimer (**1**) at 22 Å, where ω_rel_ = 1 which corresponds to “non-interacting monomers”), of the dizwitterionic-VB structures in the equilibrium geometry of the methane dimer H_3_C–H---H–CH_3_ (**1**). The relative weights of the repulsive structures, C^+^H^–^---^–^HC^+^ (Φ_6_) and C^–^H^+^---^+^HC^–^ (Φ_7_), are depicted in blue, while those of the corresponding attractive ionic structures, C^–^H^+^---^–^HC^+^ (Φ_8_) and C^+^H^–^---^+^H^–^C (Φ_9_), are depicted in red. Adapted with permission from ref (14). Copyright 2013 American Chemical Society.

To demonstrate the origins and nature of the dispersion, we plot in Figure 4 the weight changes in the dizwitterionic VB structures in the smallest molecular pair, consisting of two methane molecules H_3_C–H---H–CH_3_ (**1**).^14^ These VB weights are shown for the equilibrium H---H distance (2.425 Å) vis-à-vis a noninteracting model, wherein the H---H distance is 22 Å.^13,14^

It is apparent that Figure 4 reveals a major change in the contributions of the dizwitterionic structures of the interacting C–H---H–C bonds (vs the noninteracting two bonds at R = 22 Å). Thus, *the total weight of the two repulsive multi-ionic structures*, C^+^H^–^---^–^HC^+^ (Φ_6_) and C^–^H^+^---^+^HC^–^ (Φ_7_), decreases to ∼34% of its value in the 22 Å - separated methane molecules. By contrast, *the weight of the two stabilizing multi-ionic structures*, C^–^H^+^---^–^HC^+^ (Φ_8_) and C^+^H^–^---^+^H^–^C (Φ_9_), *increases by ∼170%* relative to the value in the 22 Å-separated methane molecules. As such, the major dispersion effect is brought about here, by the resonance of the following *two instantaneously oscillating dipoles* (C^–^H^+^---^–^HC^+^) and (C^+^H^–^---^+^H^–^C) of the interacting-moiety C–H---H–C in the H_3_C–H---H–CH_3_ dimer (**1**). Something that VB theory does not show is charge asymmetry which may be induced by symmetry-breaking due to mixing the two different sets of ionic structures. This is because the Hamiltonian in VB theory does not exhibit symmetry breaking effects for closed-shell species, and hence, it conserves the dizwitterionic symmetry of the H_3_C–H---H–CH_3_ complex (**1**).

**Figure 5:**
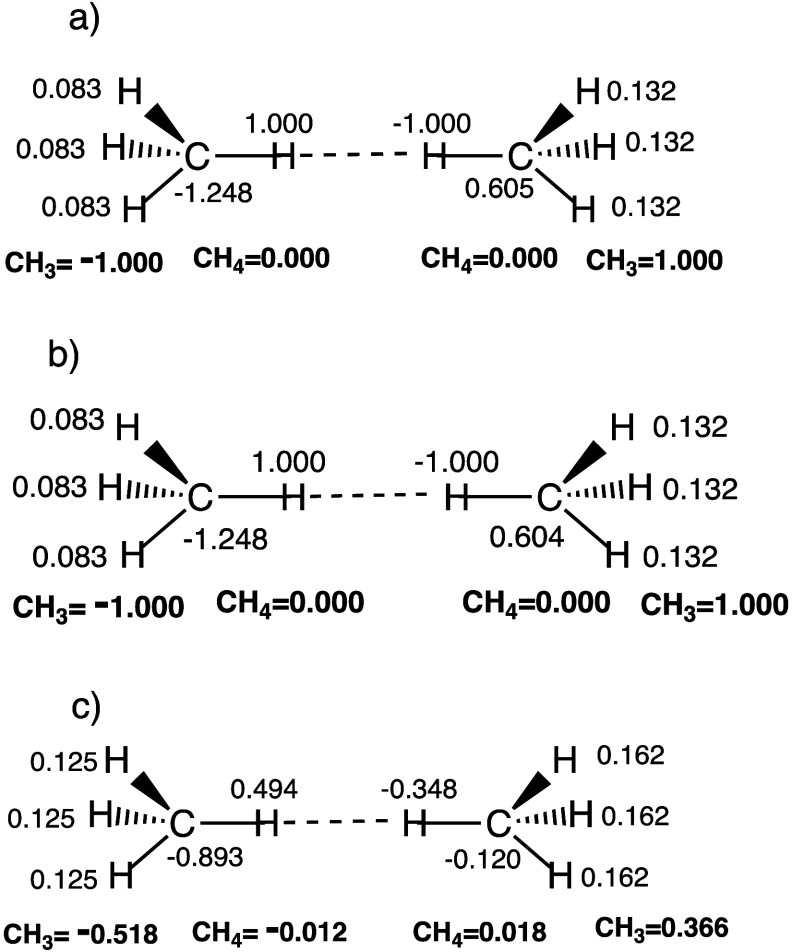
(a) Mulliken charges of the dizwitterionic VB structure, C^–^H^+^---^–^HC^+^ (Φ_8_) calculated at the VB geometry without MBD optimization. (b) Mulliken charges of the same structure calculated at the geometry optimized with MBD correction. (c) The Löwdin charges of the same VB structure calculated at the geometry optimized with MBD correction. All charges correspond to the dimer (**1**).

**Figure 6:**
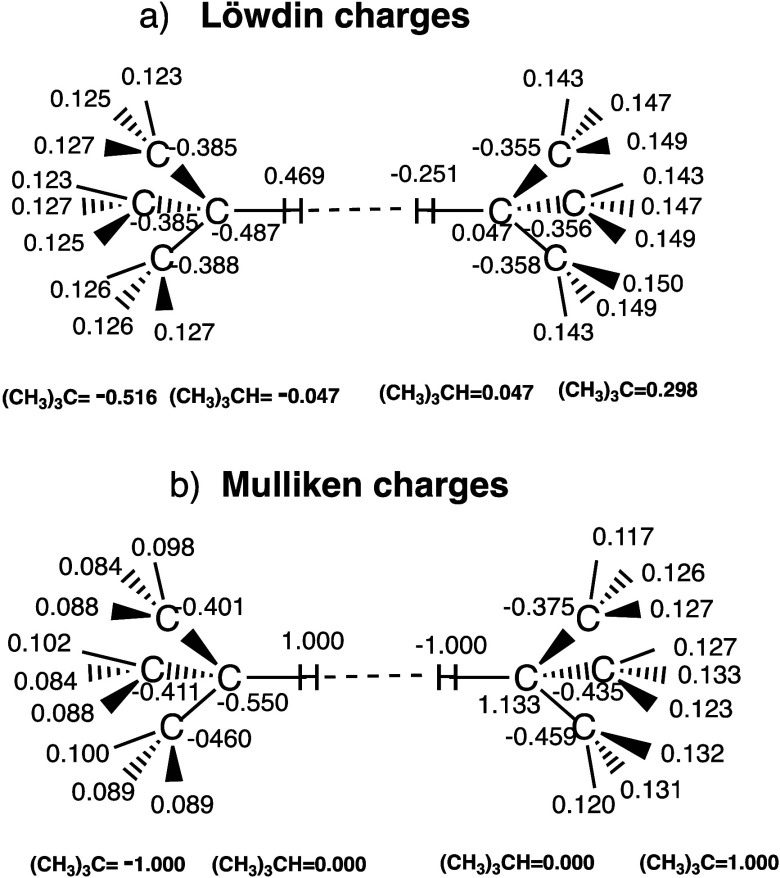
Charges on the VB structure (H_3_C)_3_C^–^ H^+^---^–^H ^+^C(CH_3_)_3_ (Φ_8_) calculated at the geometry optimized with MBD correction. Part (a) shows Löwdin charges, and part (b) Mulliken charges. All charges correspond to the dimer (**13**).

However, Figure 5 shows also charge distributions for the dizwitterionic VB structures calculated at the geometries which were optimized without (a) and with (b) MBD correction. The *R*_H–H_ distance in the case of optimization without MBD correction is 2.624 Å, vs 2.425 Å in the presence of the MBD correction. Note that the total Mulliken charges on the right and left CH_4_ molecules are zero, *but the charge distributions on the left- and right-hand CH*_3_*moieties are significantly different*, and the resulting VB structures resemble multipoles. Additionally, Figure 5c shows the Löwdin charges of the same VB structure, calculated at the geometry optimized with MBD correction. It is seen that ionic structure (c) develops a dipole moment. Apparently, the charge-asymmetry of the dizwitterionic structures in (c) reflects the mixing of the *instantaneously oscillating structures* (C^–^H^+^---^–^HC^+^) (Φ_8_) and (C^+^H^–^---^+^H^–^C) (Φ_9_), in eq 6:
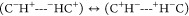
6This is further witnessed using e.g., tBu–H---H–tBu (**13**), for which the ionic VB structure in Figure 6, reflects the geometry optimized with MBD correction. The *R*_H–H_ distance in the tBu–H---H–tBu dimer (**13**) is 2.125 Å. Thus, the Mulliken charges of the dizwitterionic VB structure in tBu–H---H–tBu, calculated at the geometry optimized with MBD correction, involves delocalization of the charges. As such, the Figure reveals that for each (CH_3_)_3_CH molecule, the molecular charges on the C-H---H-C paths of the molecules alternate and are delocalized over the ensemble of atoms in the two molecules. The global charge-delocalization in Figure 6 supports the gravitational-like relationship (eq 1).

Further, in addition to the emergence of oscillating dipoles in the VB wave function (Figure 6) for tBu-H---H-tBu (**13**), there are smaller contributions of other VB structures, which involve electronic effects due to charge transfer and bond recoupling.^14^ Overall, these effects reduce the Pauli repulsion between the pair of molecules and tighten their bonding. As the original work argues,^14^ the VB mixing of the ionic structures into the covalent structures of the hydrocarbons is larger for the dimer tBu-H---H-tBu (**13**), since the ionization potential of the (CH_3_)_3_C• radical is much lower than for e.g., H_3_C•. Thus, the stabilizing ionic structures for the tBu-H---H-tBu dimer, are closer in energy to the respective covalent structures, and hence the mutual VB mixing stabilizes the dimer.^14^ Indeed, while the H---H distance in the methane dimer is 2.425 Å, in the corresponding (CH_3_)_3_C–H---H-C(CH_3_)_3_ dimer (**13**) the distance drops to 2.125 Å (see ref (14)).

**Scheme 1:**
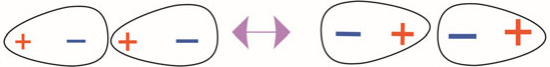
A Schematic Representation of the Resonance between the Density-Deformed Noble-Gas Atoms in the Dimer The relative charges signify formation of dipoles.

Finally, application of MBD (or D4) to the noble gas-dimers, causes rehybridization which deforms the spherical symmetry of the electron densities in the atoms. As such, for example in Ne----Ne, the dispersion interaction between the atoms is due to the 2p-3s hybridization, which creates local oscillating dipoles in the two atoms. Similarly, in Ar---Ar, 3s-3d_*z*_^2^-4p hybridization is possible, which will distort the spherical shape of the atomic electron density and induce dipole moments. This charge-density distortion brings about dispersion interactions, which are augmented by the resonance energy stabilization between the two induced dipolar wave function, as shown in Scheme 1.

In summary, all the above effects in pairs of molecules and in noble-gas dimers, create primarily oscillating dipoles in the two interacting molecules/atoms, which involve all the bonds, and which affect the ionic–covalent mixing energy (in the molecules). Similarly, the original MBD formulation which calculates the dispersion as a sum of fluctuating atomic-dipoles on all the atoms in the ensemble,^5,12,32−35^ may actually enjoy also additional covalent-ionic stabilization of the state’s wave function. Thus, while we cannot extend the VB calculations to molecules larger than the ones which we considered, the physical mechanism is extendable to the larger species in this study.

The highest dispersion energy contribution in the set of dimers in Figure 1 was found for the dodecahedrane dimer (**23**), ca. 77/81 kcal/mol (MBD/D4). The respective intermolecular portion of the CH---HC dispersion interaction, (which was calculated as a difference between the dispersion energy of dimer and sum of dispersion energies of the corresponding monomers), in the dodecahedrane pair is rather small (though larger than in (CH_4_)_2_, **1**), and most of the dispersion interaction energy (*E*_DISP_) originates in the large dispersion energies which are induced within the monomer parts (see SI, Table S6). *As we already demonstrated, our results show that the entire E*_*DISP*_*data correlates best with the gravitational relationship* (eq 1). In contrast, significantly poorer correlations were obtained with the sum of masses of the monomers in corresponding dimers, or with the summed-masses divided by the respective distances between the centers of masses.

Indeed, comparing the results for H_3_CH---HCH_3_ (**1**) vs (CH_3_)_3_CH---HC(CH_3_)_3_ (**13**) shows that the charge fluctuation in the larger dimer is delocalized to the entire ensemble of bonds/atoms of the interacting molecules. Furthermore, the effects of multiple H---H bridges between the chains appear to augment the atomic charges and transmit the charges to the entire molecules in the respective pairs (**29**–**34** in Figure 1). As such, the increased molecular size enhances the stabilization energy due to increased dispersion interactions.

Thus, the dispersion interaction involves the entire ensemble of atoms/bonds in the interacting molecules, and is the root cause of the gravitational relationship of *E*_DISP_ vs *M*_1_*M*_2_/*R*_COM-COM_ in Figures 2. Furthermore, the very large *E*_DISP_ for the dodecahedrane pair immediately suggests that dodecahedrane will form a stable solid state, which enjoys enhanced dispersion interactions for each dodecahedrane molecule with its close neighbor molecules in the solid state. This is indeed the case for dodecahedron, and the conclusion can be generalized to other cases which involve massive molecules having high dispersion energies.^13,14^ Finally, using *R*_COM-COM_ in eq 1 is preferrable (to *R*_CH-HC_) since it carries information on the extent of the contact of the monomer surfaces, which depends on the anisotropy of the monomers and topology of the interaction.^42^

## Concluding Remarks

The MBD dispersion interaction between homodimers and heterodimers of alkanes and silanes (including fluorinated derivatives, rings, noble atom-dimers, π–π interacting-dimers, 3D-objects and -cages) *obey the gravitational-like law* in eq 1. This expression emerges from the contribution of dispersion interactions by all the bonds/atoms in the molecules we studied. The Grimme-D4 correction produces similar dispersion interaction-energies, and a similar gravitational-like correlation for the same set of molecules (Figures 1, 3).

This correlation accounts for the fact, and the findings in this study, that larger molecules have generally larger dispersion stabilization. Thus, as the molecules grow in size the molecular masses grow, and so do the many-bonds/atom dispersion interactions in eq 1.

The straightforward statement of eq 1 is in agreement with the growth of *E*_DISP_ with the increased molecular sizes, noted in the present study and elsewhere.^13,14,16−25,33^ Indeed, dispersion is *a force of nature, which drives the aggregation of molecules to large entities and condensed phases*.

Does eq 1 extend all the way to macroscopic bodies? This question has major implications which deserve further exploration.

## Supporting Information Available

Different computational data calculated by means of the PBE0-MBD and PBE0-D4 methods, where the interactions depend on distances between the COMs of the monomers in the corresponding dimers; dispersion energies, masses of monomers, and Mulliken and Löwdin atomic charges; Chirgwin-Coulson weights of dizwitterionic VB structures calculated with the VBSCF and BOVB methods; dependencies of dispersion energies on *M*_1_*M*_2_/*R*_H-H_^2^, *M*_1_*M*_2_/*R*_COM-COM_^2^, *M*_1_*M*_2_/*R*_H–H_; Cartesian coordinates of dimers (PDF)

## Abbreviations

MBD: many-body dispersion
D4: dispersion method (due to S. Grimme)
DFT: density functional theory
VB: valence bond,
BOVB: breathing orbital valence bond
vdW: van der Waals
