# Supplementary material for: A Gravitational-like Relationship of Dispersion Interactions is Exhibited by 40 Pairs of Molecules and Noble Gas Atoms
Source: J Am Chem Soc. 2024 Oct 31;146(45):31198–204. doi: 10.1021/jacs.4c11211 (PMC11565636; doi:10.1021/jacs.4c11211)
Supplement: Supplementary file 1 — ja4c11211_si_001.pdf [file ja4c11211_si_001.pdf]

Supporting Information

A Gravitational-Like Relationship of Dispersion Interactions is Exhibited by 40 Pairs of Molecules and Noble Gas Atoms

David Danovich,[a(0000-0002-8730-5119)] Alexandre Tkatchenko,[b(0000-0002-1012-4854)] Santiago Alvarez,[c(0000-0002-4618-4189)] and Sason Shaik*[a(0000-0001-7643-9421)]

AFFILIATIONS: [a)] Institute of Chemistry, The Hebrew University of Jerusalem, Jerusalem 9190401, Israel. sason.shaik@gmail.com [b)] Department of Physics and Materials Science, University of Luxembourg, L-1511 Luxembourg City, Luxembourg [c)] Inorganic and Organic Chemistry Department, Universitat de Barcelona, Martí i Franquès 1-11, 08028 Barcelona, Spain.

* Author to whom correspondence should be addressed.

Table of Contents **Page**

Notes about "dihydrogen bonds" 2

Table S1. Distances between centers of masses of monomers in the corresponding dimers for MBD and D4 dispersion corrections, MBD dispersion interaction energy, D4 dispersion interaction energy and masses of monomers. 2

Table S2. Distances between centers of masses of the monomers in the corresponding dimers for MBD and D4 dispersion corrections, dispersion interaction energies E_{DISP} for MBD and D4 dispersion corrections, products of masses of monomers and $M_1xM_2/R_{com-com}$ values. 3

Table S3. Chirgwin-Coulson weights of di-zwitterionic VB structures of methane and tertiary-butyl dimers calculated with the VBSCF/6-31++G(d,p) and BOVB/6-31++G(d,p) methods. 5

Table S4. Ground-state Mulliken net atomic charges (a.u.) for the tertiary-butyl dimer calculated with PBE0-MBD/cc-pVTZ and PBE0/cc-pVTZ levels of theory. 5

Table S5. Ground-state Mulliken net atomic charges (a.u.) of dodecahedrane dimer calculated with the PBE-MBD/cc-pVTZ level of theory. 6

Figure S1. Mulliken atomic charges (a.u.) of dodecahedrane dimer. 8

Figure S2. Mulliken and Löwdin charges of the ionic valence bond (VB) structures Φ_8 and Φ_9 of dimer **1** calculated with VBSCF,BOVB/6-31++G(d,p) methods. 9

Figure S3. Mulliken and Löwdin charges of ionic valence bond (VB) structures Φ_9 of dimer **14** calculated with VBSCF,BOVB/6-31++G(d,p) methods. 10

Figure S4. Mulliken and Löwdin charges of ionic valence bond (VB) structures Φ_8 of dimer **14** calculated with VBSCF/6-31++G(d,p) method. 11

Figure S5. Mulliken and Löwdin charges of ionic valence bond (VB) structures Φ_8 and Φ_9 of dimer **14** calculated by BOVB/6-31++G(d,p) method with 2 structures. 12

Table S6. MBD dimers dispersion interaction energy, MBD monomers dispersion interaction energy and ΔE_{DISP} values 13

Table S7. Distances between centers of masses of the monomers in the dimers calculated with MBD correction, MBD dispersion interaction energy, mass of monomers, sum of mass of monomers and $M_1+M_2/R_{com-com}$ values 14

Figure S6. Dependence of the total dispersion energy calculated with 15

Tkachenko's MBD correction on M_1+M_2/$R_{COM-COM}$, on M_1+M_2 and on M_1xM_2 for the entire set of the dimers.
Full citation for Ref. 29. 16
Table S8. Bond lengths between closest H atoms of the dimers calculated with MBD correction, MBD and D4 dispersion interaction energies. 17
Table S9. Square of bond lengths between closest H atoms of the dimers (R^2_{H-H}), square of distances between the respective centers of masses ($R^2_{com-com}$), $E_{DISP,MBD}$ and M_1xM_2/R^2_{H-H} and M_1xM_2/$R^2_{com-com}$ values 17
Figure S7. Dependence of the dispersion interaction energy on M_1xM_2/R^2_{H-H} (a) for 26 dimers and on M_1xM_2/$R^2_{COM-COM}$ (b) for 40 dimers 19
Table S10. Bond lengths between closest H atoms of the dimers, dispersion interaction energies of dimers, product of masses of monomers and M^2/R_{H-H} 19
Figure S8. Dependence of the dispersion interaction energy on M_1xM_2/R_{H-H} for 26 dimers 20
Cartesian Coordinates of investigated dimers in Å optimized by PBE0-MBD/cc-pVTZ 20

Notes about "dihydrogen bonds"

The term "dihydrogen bonds" was proposed by Crabtree et al. (*J. Am. Chem. Soc.* **1995**, 117, 12875-12876) for the interactions between two hydrogen atoms of opposite polarity, i.e., one with protonic and another with hydridic character. On the other hand, Matta et al.(*J. Phys. Chem. A* , **2011**, 115, 12451-12458) point out that «the non electrostatic interaction between two closed-shell neutral (or close to neutral) hydrogen atos has been termed hydrogen-hydrogen (or H-H) interaction to distinguish it from the dihydrogen bond», providing abundant references for the two cases. We have also referred to this case as "homopolar H···H interactions" or "homopolar dihydrogen interactions": Echeverría, J. et al. *Phys. Chem. Chem. Phys.* **2017**, 19, 28044-28055; Echeverría, J.; Alvarez, S. *Chem. Sci.* **2023**, 14, 11647-11688.

Table S1. Distances between centers of masses of monomers in the corresponding dimers ($R_{com-com}$, in Å) calculated with MBD and D4 dispersion corrections, MBD dispersion interaction energies[a] ($E_{DISP,MBD}$, in au), D4 dispersion interaction energies[a] ($E_{DISP,D4}$, in au) and masses of monomers (M_1, M_2 in amu)[b]

	$R_{com-com}$ MBD	$R_{com-com}$ D4	$E_{DISP,MBD}$	$E_{DISP,D4}$	M_1(amu)	M_2(amu)
1	4.6025	4.6025	-0.00208376	-0.0017843	16.04236	
2	5.3501	5.3502	-0.00217864	-0.0020411	34.0330	
3	5.7358	5.7358	-0.00221213	-0.0024345	52.02258	
4	5.5752	5.5751	-0.00265807	-0.0030396	32.1170	
5	4.0619	4.0619	-0.00232540	-0.0020229	16.04236	
6	3.6367	3.6367	-0.00262624	-0.0022921	16.04236	
7	5.0789	5.1038	-0.00549617	-0.0050074	30.06904	
8	5.8529	5.8525	-0.00592966	-0.0068412	58.299	
9	6.0263	6.0261	-0.00670277	-0.0089262	62.218	

10	6.2911	6.2911	-0.00994873	-0.0093020	44.09572	
11	6.7596	6.7596	-0.01051204	-0.0115907	60.1710	
12	6.8738	6.8738	-0.01071294	-0.0136797	77.3405	
13	5.0887	5.0887	-0.01028948	-0.0096433	44.09572	
14	5.0432	5.0669	-0.01641880	-0.0156177	58.12240	
15	6.3857	6.3857	-0.00726311	-0.0075335	52.07536	
16	7.1261	7.1261	-0.02742006	-0.0292114	104.15072	
17	7.3228	7.3227	-0.05584619	-0.0560462	136.238	
18	7.2144	7.2144	-0.01492743	-0.0190080	78.11184	
19	6.8083	6.8083	-0.01573593	-0.0168938	78.11184	
20	7.3773	7.3776	-0.04170417	-0.0435226	130.901	
21	7.7377	7.7378	-0.05575758	-0.0579044	156.2237	
22	8.1498	8.1498	-0.06796890	-0.0708924	182.2610	
23	8.4719	8.4720	-0.12338109	-0.1293960	260.3728	
24	6.5801	6.5801	-0.01150023	-0.0121986	78.11184	52.07536
25	5.5982	5.5978	-0.00593488	-0.0054693	44.09572	16.04236
26	6.1510	6.1509	-0.01880351	-0.0193925	44.09572	104.15072
27	7.1169	7.1168	-0.00856597	-0.0111620	77.3405	62.218
28	5.1344	5.1344	-0.01080644	-0.0101855	58.12240	30.06904
29	4.7987	4.7986	-0.01068687	-0.0100787	44.09572	
30	5.4266	5.4265	-0.01012972	-0.0095448	44.09572	
31	4.8789	4.8771	-0.02129390	-0.0202527	73.0063	
32	5.6750	5.6740	-0.02089291	-0.0198782	73.0063	
33	4.9441	4.9437	-0.03221595	-0.0307239	100.20194	
34	5.8017	5.8017	-0.03208576	-0.0305663	100.20194	
35	4.0594	4.0594	-0.01765028	-0.0226434	78.11184	
36	4.0623	4.0622	-0.00370992	-0.0041835	28.05316	
37	4.2555	4.2449	-0.00995313	-0.01249564	78.11184	28.05316
38	3.0525	3.0738	-0.00008740	-0.00007678	20.1797	
39	3.6638	3.6650	-0.00028862	-0.00042516	39.9480	
40	2.7400	2.7975	-0.00001016	-0.00002743	4.00260	

a) Calculated with cc-pVTZ basis set. b) Calculated with the following masses of the corresponding atoms: C=12.0107 amu, H=1.00794 amu, F=18.998 amu, Si=28.085 amu, He=4.0026 amu, Ne=20.1797 amu, Ar=39.9480 amu.

Table S2. Distances between centers of masses in the corresponding dimers ($R_{com\text{-}com}$, in Å) calculated with MBD and D4 dispersion corrections, dispersion interaction energies (E_{DISP}, kcal/mol)[a] for MBD and D4 dispersion corrections, products of monomers masses[b] (M_1xM_2, in amu) and $M_1xM_2/R_{com\text{-}com}$ values (when the MBD/$R_{com\text{-}com}$ and D4/$R_{com\text{-}com}$ values are not coincident, the MBD value is given first and the D4 value below it).

	$R_{com\text{-}com}$ MBD	$R_{com\text{-}com}$ D4	$E_{DISP,MBD}$	$E_{DISP,D4}$[c]	M_1xM_2	$M_1xM_2/R_{com\text{-}com}$[d]
1	4.6025	4.6025	-1.31	-1.12 (-1.12)	257.357314	55.91685
2	5.3501	5.3502	-1.37	-1.28 (-1.28)	1158.24509	216.49034<br>216.4863183
3	5.7358	5.7358	-1.39	-1.53 (-1.53)	2706.34883	471.83459
4	5.5752	5.5751	-1.67	-1.91 (-1.91)	1031.50169	185.01609<br>185.019406

5	4.0619	4.0619	-1.46	-1.27	257.357314	63.35885
6	3.6367	3.6367	-1.65	-1.44	257.357314	70.76671
7	5.1038	5.1038	-3.45	-3.14	904.147167	177.151763
8	5.8529	5.8525	-3.73	-4.29	3398.77340	580.69904<br>580.738727
9	6.0263	6.0261	-4.21	-5.60	3871.07952	642.36422<br>642.3855429
10	6.2911	6.2911	-6.24	-5.84	1944.43252	309.0767
11	6.7596	6.7596	-6.60	-7.27	3620.54924	535.61590
12	6.8738	6.8738	-6.72	-8.58 (-8.58)	5981.55294	870.19595
13	5.0887	5.0887	-6.46	-6.05 (-6.05)	1944.43252	382.1079
14	5.0432	5.0669	-10.30	-9.80 (-9.80)	3378.21338	669.8551<br>666.7219365
15	6.3857	6.3857	-4.55	-4.73 (-4.73)	2711.84312	424.6744
16	7.1261	7.1261	-17.21	-18.33 (-18.3)	10847.3724	1522.2032
17	7.3228	7.3227	-35.04	-35.17 (-35.2)	18560.79264	2534.6579<br>2534.692482
18	7.2144	7.2144	-9.37	-11.93 (-11.9)	6101.45330	845.7326
19	6.8083	6.8083	-9.87	-10.60 (-10.6)	6101.45330	896.1787
20	7.3773	7.3776	-26.17	-27.31 (-27.3)	17135.0718	2322.6752<br>2322.580758
21	7.7377	7.7378	-34.99	-36.34 (-36.3)	24405.84444	3154.1472<br>3154.106392
22	8.1498	8.1498	-42.65	-44.49 (-44.5)	33219.07212	4076.0598
23	8.4719	8.4720	-77.42	-81.20 (-81.20)	67793.99498	8002.2185<br>8002.124053
24	6.5801	6.5801	-7.22	-7.65	4067.702188	618.18243
25	5.5982	5.5978	-3.72	-3.43	707.3994147	126.36194<br>126.3709698
26	6.1510	6.1509	-11.80	-12.17	4592.600987	746.642983<br>746.65512215
27	7.1169	7.1168	-5.38	-7.00	4811.971229	676.133040<br>676.14254
28	5.1344	5.1344	-6.78	-6.39	1747.684771	340.387342
29	4.7987	4.7986	-6.71	-6.32 (-6.32)	1944.423703	405.19801<br>405.206457
30	5.4266	5.4265	-6.36	-5.99 (-5.99)	1944.423703	358.313438<br>358.320041
31	4.8789	4.8771	-13.36	-12.71 (-12.7)	5329.91984	1092.442936<br>1092.846126
32	5.6750	5.6740	-13.11	-12.47 (-12.5)	5329.91984	939.1929233<br>939.3584491
33	4.9441	4.9437	-20.22	-19.28 (-19.3)	10040.42878	2030.789988<br>2030.954301
34	5.8017	5.8016	-20.13	-19.18 (-19.2)	10040.42878	1730.601165<br>1730.630995
35	4.0594	4.0594	-11.076	-14.21 (-14.2)	6101.459548	1503.044674<br>1503.044674
36	4.0623	4.0622	-2.328	-2.63	786.979786	193.7290692

						193.7324076
37	4.2555	4.2449	-6.245	-7.84 (-7.84)	2191.283945	514.9298427<br>516.215681
38	3.0524	3.0738	-0.055	-0.048	407.2202921	133.404627<br>132.481063
39	3.6638	3.6650	-0.181	-0.27	1595.842704	435.5703652<br>435.4277501
40	2.7400	2.7400	-0.0064	-0.0172	16.02080678	5.847

a) Calculated with cc-pVTZ basis set. b) Calculated with the following masses of the corresponding atoms: C=12.0107 amu, H=1.00794 amu, F=18.998 amu, Si=28.085 amu. He=4.0026 amu, Ne=20.1797 amu, Ar=39.9480 amu. c) In parentheses are the results for pairwise-only D4 calculations. d) The numbers in the second line corresponds to D4 result

Table S3. Chirgwin-Coulson weights (ω) of the VB structures of methane and tertiary-butyl dimers calculated with VBSCF/6-31++G(d,p) and BOVB/6-31++G(d,p) methods

	H_3C-H---H-CH_3		$(H_3C)_3C$-H---H-$C(CH_3)_3$	
structure	ω_{VBSCF}	ω_{BOVB}	ω_{VBSCF}	ω_{BOVB}
Φ_1 ($C^{\cdot}$ $^{\cdot}H$ --- $H^{\cdot}$ $^{\cdot}C$)	0.544	0.627	0.543	0.630
Φ_8 (C^{+} ^{-}H --- H^{+} ^{-}C)	0.019	0.080	0.022	0.064
Φ_9 (C^{-} ^{+}H --- H^{-} ^{+}C)	0.019	0.080	0.022	0.064
Φ_6 (C^{+} ^{-}H --- H^{-} ^{+}C)	0.004	0.029	0.006	-0.022
Φ_7 (C^{-} ^{+}H --- H^{+} ^{-}C)	0.026	0.020	0.019	0.027

Table S4. Ground-state Mulliken net atomic charges (au) of the tertiary-butyl dimer calculated with PBE0-MBD/cc-pVTZ and PBE0/cc-pVTZ levels of theory

Atom	PBE0 Dimer	PBE0-MBD dimer	PBE0-MBD monomer
1 C	-0.077188	**-0.077538**	-0.053505
2 H	0.110160	**0.110385**	0.085687
3 C	-0.293823	-0.293969	-0.291303
4 H	0.110159	**0.110385**	
5 C	-0.077188	**-0.077538**	
6 C	-0.293823	-0.293969	
7 H	0.090132	0.090183	0.089330
8 H	0.096326	0.096386	0.095659
9 H	0.096362	0.096422	0.095691
10 H	0.090133	0.090183	
11 H	0.096362	0.096422	
12 H	0.096326	0.096386	
13 C	-0.292778	-0.292836	-0.290874
14 H	0.089849	0.089928	0.089311
15 H	0.095696	0.095752	0.095160
16 H	0.096251	0.096258	0.095623
17 C	-0.292778	-0.292836	
18 H	0.095696	0.095752	

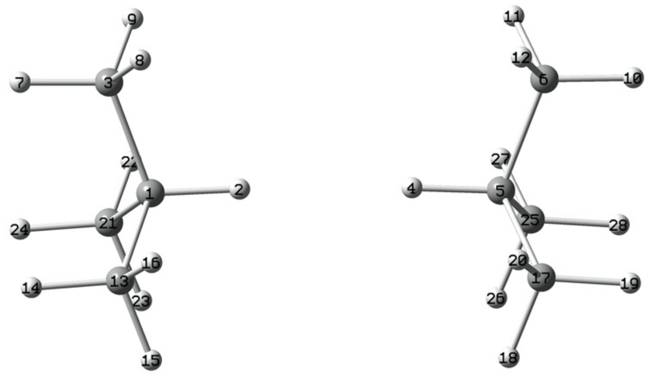

19 H	0.089849	0.089928	
20 H	0.096251	0.096258	
21 C	-0.292788	-0.292967	-0.290946
22 H	0.095919	0.096011	0.095347
23 H	0.095943	0.095993	0.095426
24 H	0.089941	0.089993	0.089366
25 C	-0.292788	-0.292967	
26 H	0.095943	0.095993	
27 H	0.095919	0.096011	
28 H	0.089941	0.089993	

Sum of atomic charges = 0.000000

Table S5. Ground-state Mulliken net atomic charges (au) of dodecahedrane dimer calculated with PBE-MBD/cc-pVTZ level of theory

Atom	PBE0-MBD Dimer	PBE0-MBD monomer
1 C	−0.088538	−0.088573
2 C	−0.090281	−0.089371
3 H	0.088580	0.088601
4 H	0.088579	0.088630
5 C	−0.102629	−0.088649
6 C	−0.091302	−0.090707
7 H	0.089235	0.088524
8 H	0.088417	0.088519
9 C	−0.089399	−0.088612
10 C	−0.091426	−0.090627
11 H	0.088554	0.088600
12 H	0.088465	0.088513
13 C	−0.088865	−0.088540
14 C	−0.089227	−0.089344
15 H	0.088602	0.088594
16 H	0.088664	0.088656
17 C	−0.088373	−0.087614
18 H	0.088501	0.088564
19 C	−0.087969	−0.087645
20 H	0.088585	0.088577
21 C	−0.087549	−0.087589
22 H	0.088552	0.088569
23 C	−0.101661	−0.087796
24 H	0.089173	0.088514
25 C	−0.098735	−0.085430
26 H	0.089330	0.088794
27 C	−0.088472	−0.088526
28 H	0.088664	0.088675
29 C	−0.085633	−0.085314
30 H	0.088848	0.088841
31 C	−0.089476	−0.088597
32 H	0.088577	0.088668

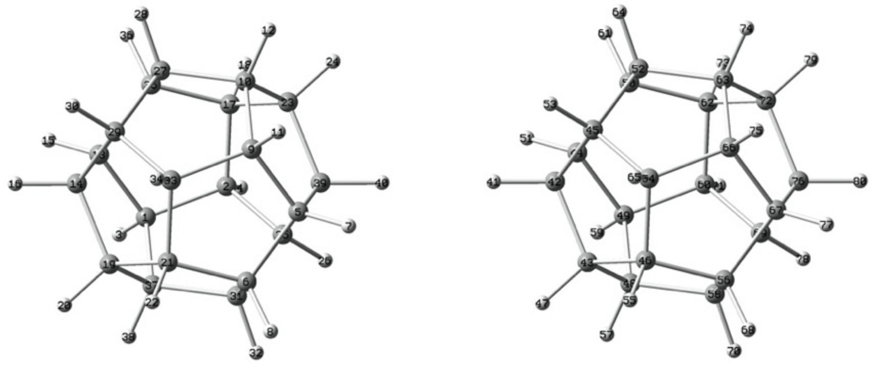

33	C	−0.089342	−0.089361
34	H	0.088627	0.088642
35	C	−0.090614	−0.090686
36	H	0.088506	0.088523
37	C	−0.090520	−0.090593
38	H	0.088501	0.088518
**39**	**C**	**−0.089706**	**−0.088835**
**40**	**H**	**0.134763**	**0.088883**
**41**	**H**	**0.134743**	
**42**	**C**	**−0.089654**	
43	C	−0.101702	
44	C	−0.102560	
45	C	−0.098747	
46	C	−0.088329	
47	H	0.089183	
48	C	−0.091366	
49	C	−0.089470	
50	C	−0.091373	
51	H	0.089223	
52	C	−0.089427	
53	H	0.089328	
54	C	−0.090341	
55	H	0.088498	
56	C	−0.090669	
57	H	0.088466	
58	C	−0.088469	
59	H	0.088555	
60	C	−0.089359	
61	H	0.088418	
62	C	−0.087528	
63	C	−0.090449	
64	H	0.088571	
65	H	0.088583	
66	C	−0.088621	
67	C	−0.088785	
68	H	0.088506	
69	C	−0.085638	
70	H	0.088668	
71	H	0.088621	
72	C	−0.088007	
73	H	0.088551	
74	H	0.088496	
75	H	0.088586	
76	C	−0.089204	
77	H	0.088594	
78	H	0.088848	
79	H	0.088591	
80	H	0.088668	

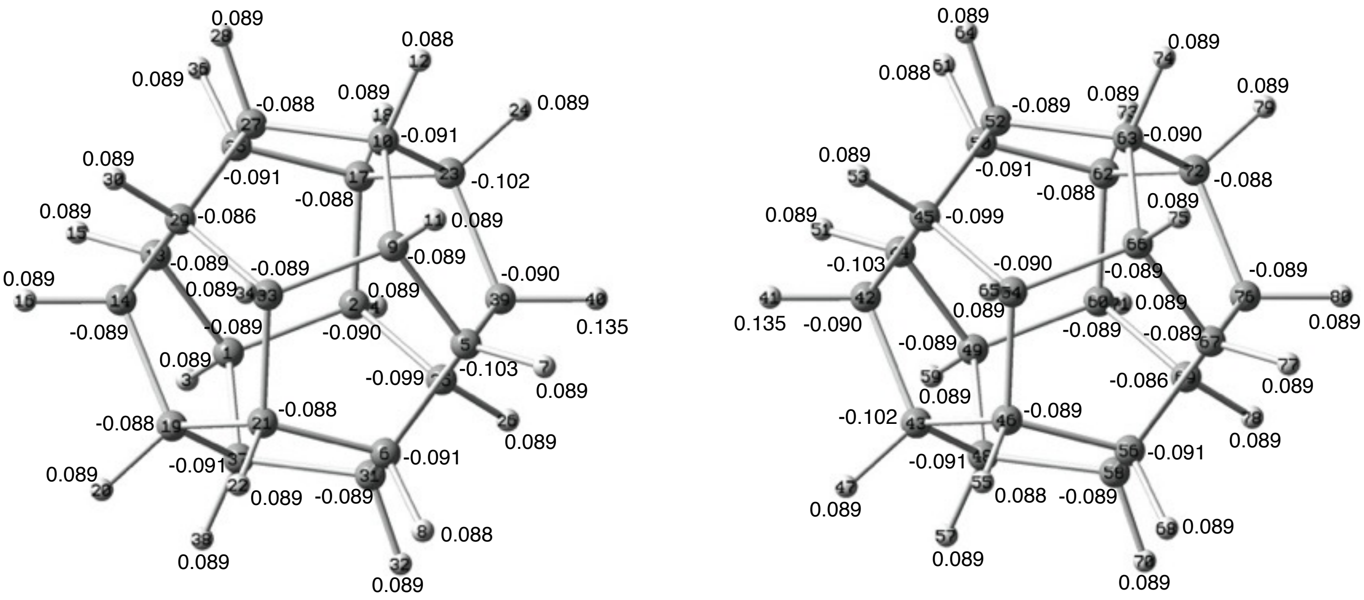

Figure S1. Mulliken atomic charges (au) in the dodecahedrane dimer.

Ionic structure Φ9 used for VBSCF calculations

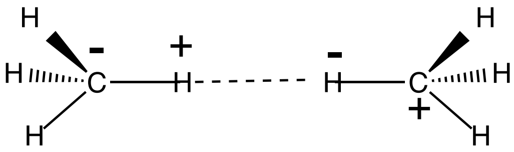

Lowdin charges at geometry optimized with MBD corrections (H-H=2.425Å)

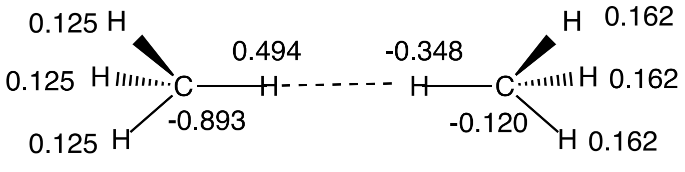

CH_3=-0.518 CH_4=-0.012 CH_4=0.018 CH_3=0.366

Mulliken charges at geometry optimized with MBD corrections (H-H=2.425Å)

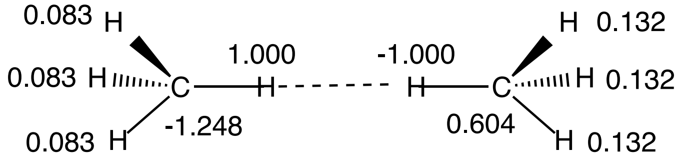

CH_3=-1.000 CH_4=0.000 CH_4=0.000 CH_3=1.000

Two ionic structures (Φ8 and Φ9) used for VBSCF calculations. In parenthesises BOVB

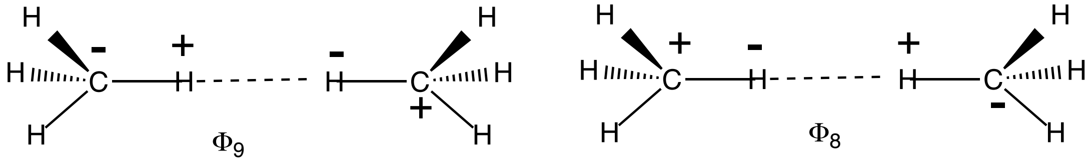

Lowdin charges at geometry optimized with MBD corrections (H-H=2.425Å)

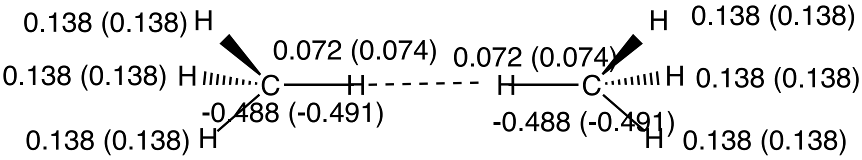

CH_3=-0.518 CH_4=-0.012 CH_4=0.018 CH_3=0.366

Mulliken charges at geometry optimized with MBD corrections (H-H=2.425Å)

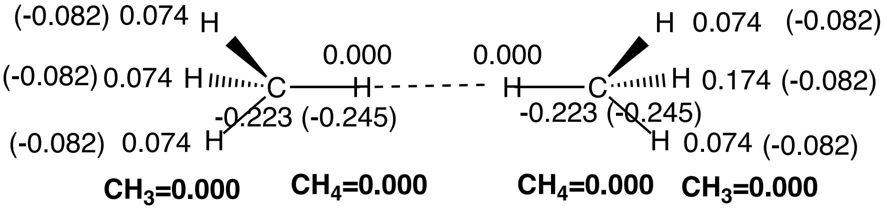

CH_3=0.000 CH_4=0.000 CH_4=0.000 CH_3=0.000

Figure S2. Mulliken and Löwdin charges of the ionic valence bond (VB) structures Φ8 and Φ9 of dimer **1** calculated by VBSCF/6-31++G(d,p) and BOVB/6-31++G(d,p) methods

Ionic structure Φ_9 used for VBSCF calculations

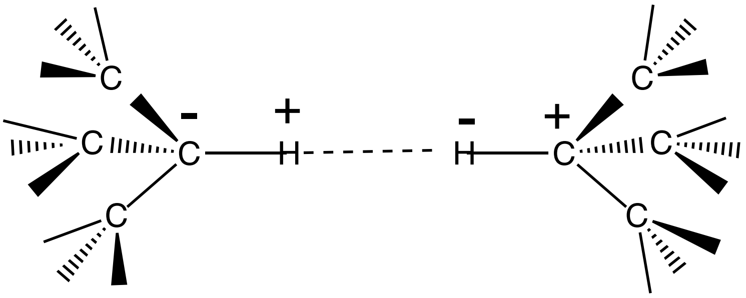

Lowdin charges at geometry optimized with MBD corrections (H-H=2.125Å)

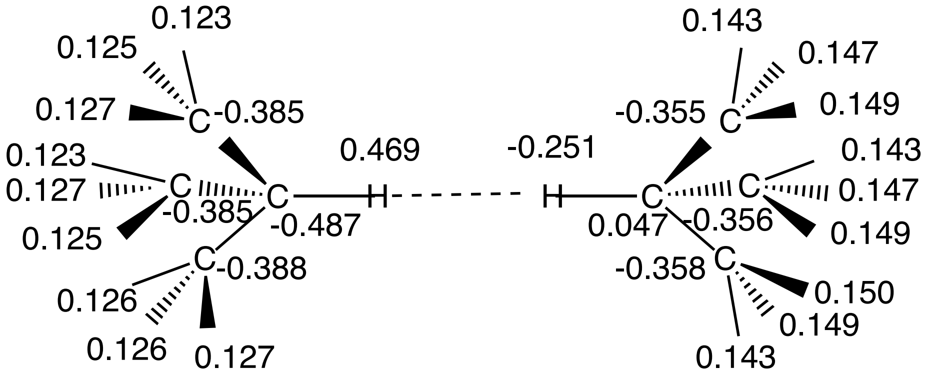

$(CH_{3)3}C$=-0.516 **$(CH_3)_3CH$=-0.047** **$(CH_3)_3CH$=0.047** **$(CH_3)_3C$=0.298**

Mulliken charges at geometry optimized with MBD corrections (H-H=2.125Å)

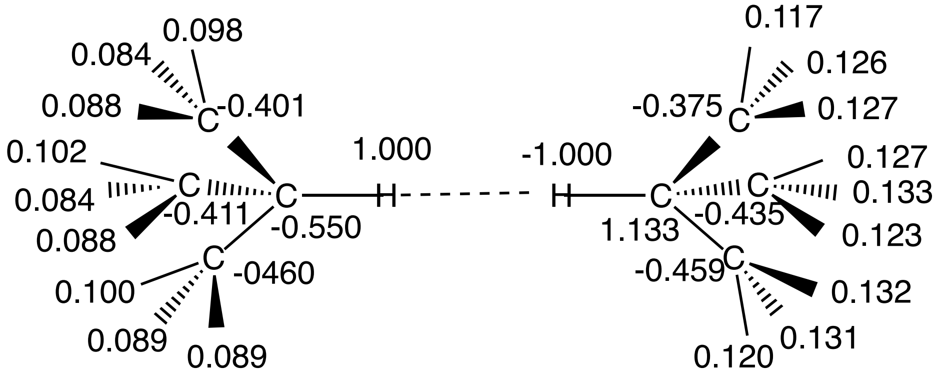

$(CH_{3)3}C$=-1.000 **$(CH_3)_3CH$=0.000** **$(CH_3)_3CH$=0.000** **$(CH_3)_3C$=1.000**

Figure S3. Mulliken and Löwdin charges of ionic valence bond (VB) structures Φ_9 of dimer **14** calculated by VBSCF/6-31++G(d,p) method

Ionic structure Φ_8 used for VBSCF calculations

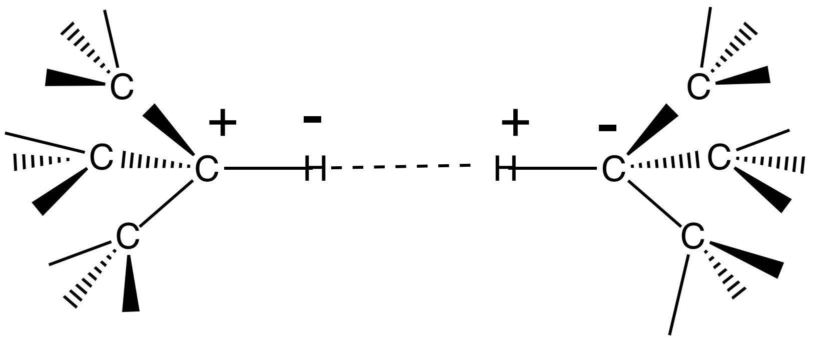

Lowdin charges at geometry optimized with MBD corrections (H-H=2.125Å)

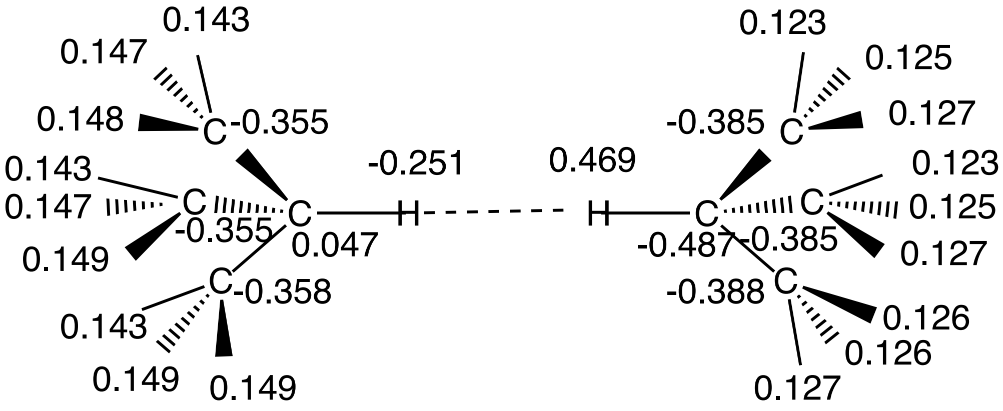

(CH_3)$_3$C=0.298 **(CH_3)$_3$CH=0.047** **(CH_3)$_3$CH=-0.049** **(CH_3)$_3$C=-0.518**

Mulliken charges at geometry optimized with MBD corrections (H-H=2.125Å)

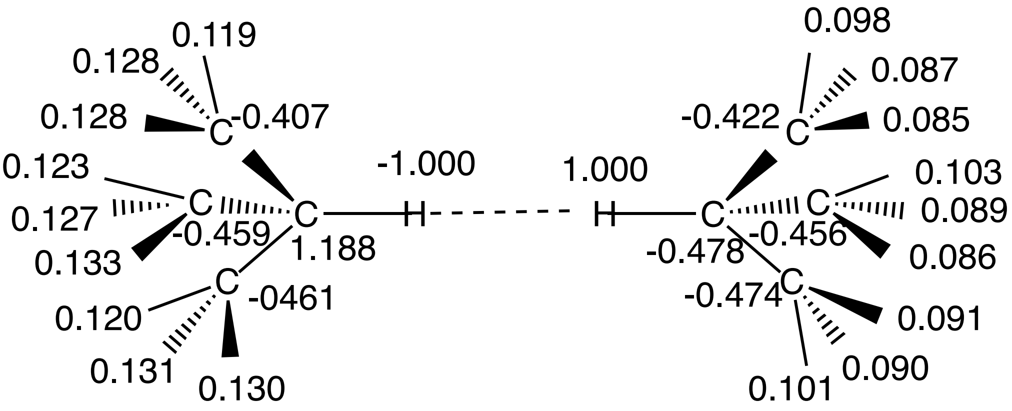

(CH_3)$_3$C= 1.000 **(CH_3)$_3$CH=0.000** **(CH_3)$_3$CH=0.000** **(CH_3)$_3$C= -1.000**

Figure S4. Mulliken and Löwdin charges of ionic valence bond (VB) structures Φ_8 of dimer **14** calculated by VBSCF/6-31++G(d,p) method

Ionic structures Φ_8 and Φ_9 used for VBSCF calculations

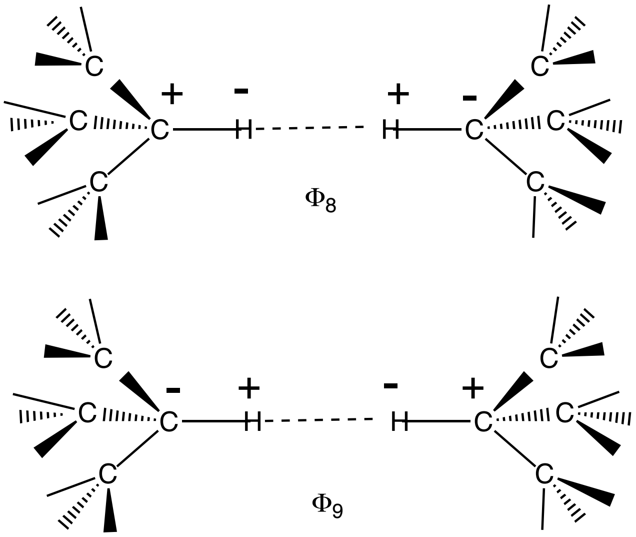

Lowdin charges at geometry optimized with MBD corrections (H-H=2.125Å)

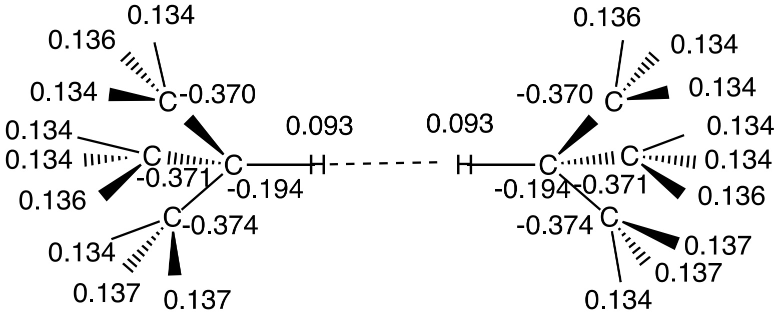

$(CH_{3)3}C$=-0.093 **$(CH_3)_3CH$=0.000** **$(CH_3)_3CH$=0.000** **$(CH_3)_3C$=-0.093**

Mulliken charges at geometry optimized with MBD corrections (H-H=2.125Å)

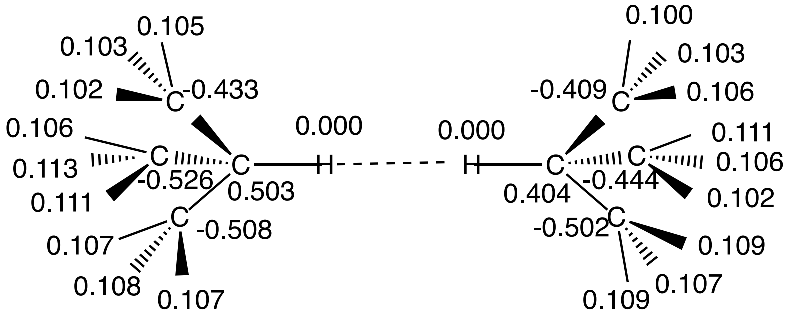

$(CH_{3)3}C$=-0.002 **$(CH_3)_3CH$=0.000** **$(CH_3)_3CH$=0.000** **$(CH_3)_3C$=0.002**

Figure S5. Mulliken and Löwdin charges of ionic valence bond (VB) structures Φ_8 and Φ_9 of dimer **14** calculated by BOVB/6-31++G(d,p) method with 2 structures.

Table S6. MBD dispersion interaction energy in dimers (E_{DISP} (dimers), in au and kcal/mol),[a] MBD dispersion energy in monomers (E_{DISP} (monomer), in au),[a] $\Delta E_{DISP} = E_{DISP}$ (dimer) – $2xE_{DISP}$ (monomer) (in au and kcal/mol).

	$E_{DISP,MBD}$ (dimers) au	$E_{DISP,MBD}$ (dimers) kcal/mol	$E_{DISP,MBD}$ (monomer) au	ΔE_{DISP}, au	ΔE_{DISP} kcal/mol	ΔE_{DISP}[e] au
1	-0.00208376	-1.31	-0.000828028	-0.0004277	-0.268	-0.0005596
2	-0.00217864	-1.37	-0.000884155	-0.0004103	-0.258	-0.0004293
3	-0.00221213	-1.39	-0.000959501	-0.0002931	-0.183	-0.0001537
4	-0.00265807	-1.67	-0.001083450	-0.0004912	-0.308	-0.0006738
5	-0.00232540	-1.46	-0.000827732	-0.0006699	-0.420	-0.0008392
6	-0.00262624	-1.65	-0.000827946	-0.0009703	-0.609	-0.0012503
7	-0.00549617	-3.45	-0.002411496	-0.0006950	-0.436	
8	-0.00592966	-3.73	-0.002643447	-0.0006428	-0.403	
9	-0.00670277	-4.21	-0.002917356	-0.0008681	-0.545	
10	-0.00994873	-6.24	-0.004579206	-0.0007903	-0.496	
11	-0.01051204	-6.60	-0.004887285	-0.0007375	-0.463	
12	-0.01071294	-6.72	-0.004884749	-0.0009434	-0.592	
13	-0.01028948	-6.46	-0.004576332	-0.0012569	-0.789	
14	-0.01641880	-10.30	-0.007353235	-0.0017124	-1.075	-0.0015207
15	-0.00726311	-4.55	-0.003285428	-0.0006923	-0.435	
16	-0.02742006	-17.21	-0.013136080	-0.0011479	-0.720	
17	-0.05584619	-35.04	-0.026858773	-0.0021288	-1.336	
18	-0.01492743	-9.37	-0.006960942	-0.0010552	-0.662	
19	-0.01573593	-9.87	-0.007395117	-0.0009457	-0.593	
20	-0.04170417	-26.17	-0.020110266	-0.0014836	-0.931	
21	-0.05575758	-34.99	-0.026968242	-0.0018211	-1.143	
22	-0.06796890	-42.65	-0.032891461	-0.0021860	-1.372	
23	-0.12338109	-77.42	-0.06020703[b]<br>-0.06020233[c]	-0.0029670[b]<br>-0.0029717[c]	-1.862[b]<br>-1.865[c]	
24	-0.01150023	-7.22	-0.010663566[d]	-0.0008367	-0.525	
25	-0.00593488	-3.72	-0.005402137[d]	-0.0005327	-0.334	
26	-0.01880351	-11.80	-0.016696186[d]	-0.0021073	-1.322	
27	-0.00856597	-5.38	-0.007789613[d]	-0.0007764	-0.487	
28	-0.01080644	-6.78	-0.009750133[d]	-0.0010564	-0.663	
29	-0.01068687	-6.71	-0.004565975	-0.0015549	-0.976	
30	-0.01012972	-6.36	-0.004565115	-0.0009995	-0.627	
31	-0.02129390	-13.36	-0.009280634	-0.0023316	-1.463	
32	-0.02089291	-13.11	-0.009288666	-0.0023156	-1.453	
33	-0.03221595	-20.22	-0.014105688	-0.0040046	-2.513	
34	-0.03208576	-20.13	-0.014106853	-0.0038721	-2.430	
35	-0.01765028	-11.076	-0.006958157	-0.0037340	-2.343	
36	-0.00370992	-2.328	-0.001397514	-0.0009149	-0.574	
37	-0.00995313	-6.245	-0.008347914[d]	-0.0016052	-1.007	
38	-0.00008740	-0.055				
39	-0.00028862	-0.181				
40	-0.00001016	-0.0064				

a) Calculated with cc-pVTZ basis set. b) Dispersion energy of one monomer was calculated and was multiplied by 2 in order to get the dispersion energy of the two monomers. c) Dispersion energy of each monomer in dimer **23** was calculated and total dispersion energy of both monomers was obtained as a sum of the dispersion energy of the monomers. d) E_{DISP} of the sum of the monomers. e) Calculated by DLPNO-CCSD(T) LED approach of Neese (Refs. 23-25 in the manuscript).

Table S7. Distances between centers of masses of the monomers in the dimers ($R_{com\text{-}com}$, in Å) calculated with MBD correction, MBD dispersion interaction energy ($E_{DISP,MBD}$) in kcal/mol),[a] masses of monomers (M_1, M_2, in amu),[b] sum of mass of monomer (M_1+M_2, in amu) and (M_1+M_2)/$R_{com\text{-}com}$ values

	$R_{com\text{-}com}$ MBD	$E_{DISP,MBD}$	M_1(amu)	M_2(amu)	M_1+M_2	(M_1+M_2)/$R_{com\text{-}com}$
1	4.6025	-1.31	16.04236		32.08472	6.971150
2	5.3501	-1.37	34.0330		68.066	12.722379
3	5.7358	-1.39	52.02258		104.04516	18.1396074
4	5.5752	-1.67	32.1170		64.234	11.5213804
5	4.0619	-1.46	16.04236		32.08472	7.89894384
6	3.6367	-1.65	16.04236		32.08472	8.82248192
7	5.0789	-3.45	30.06904		60.13808	11.8407687
8	5.8529	-3.73	58.299		116.598	19.9214065
9	6.0263	-4.21	62.218		124.436	20.6488227
10	6.2911	-6.24	44.09572		88.19144	14.0184451
11	6.7596	-6.60	60.1710		120.342	17.8031245
12	6.8738	-6.72	77.3405		154.681	22.5029823
13	5.0887	-6.46	44.09572		88.19144	17.3308389
14	5.0432	-10.30	58.12240		116.2448	23.0498096
15	6.3857	-4.55	52.07536		104.15072	15.3099926
16	7.1261	-17.21	104.15072		208.30144	29.230777
17	7.3228	-35.04	136.238		272.4760	37.2092642
18	7.2144	-9.37	78.1118		156.22360	21.654413
19	6.8083	-9.87	78.1118		156.22360	22.9460511
20	7.3773	-26.17	130.901		261.802	35.4875090
21	7.7377	-34.99	156.2237		312.4474	40.3798803
22	8.1498	-42.65	182.2610		364.5220	44.7277234
23	8.4719	-77.42	260.3728		520.7456	61.4673922
24	6.5801	-7.22	78.11184	52.07536	130.1872	19.7849881
25	5.5982	-3.72	44.09572	16.04236	60.13808	10.7423958
26	6.1510	-11.80	44.09572	104.15072	148.24644	24.1011933
27	7.1169	-5.38	77.3405	62.218	139.5585	19.6094507
28	5.1344	-6.78	58.12240	30.06904	88.19144	17.1765815
29	4.7987	-6.71	44.09572		88.19144	18.3781941
30	5.4266	-6.36	44.09572		88.19144	16.2516935
31	4.8789	-13.36	73.0063		146.0126	29.9273607
32	5.6750	-13.11	73.0063		146.0126	25.7290925
33	4.9441	-20.22	100.20194		200.40388	40.5339455
34	5.8017	-20.13	100.20194		200.40388	34.5422687
35	4.0594	-11.076	78.11184		156.22368	38.4844263

36	4.0623	-2.328	28.05316		56. 10632	13.8114664
37	4.2555	-6.245	78.11184	28.05316	106.165	24.9477147
38	3.0524	-0.055	20.1797		40.3594	13.2221858
39	3.6638	-0.181	39.9480		79.896	21.8068672
40	2.7400	-0.0064	4.00260		8.0052	2.92160584

a) Calculated with cc-pVTZ basis set. b) Calculated with the following masses of the corresponding atoms: C=12.0107 amu, H=1.00794 amu, F=18.998 amu, Si=28.085 amu

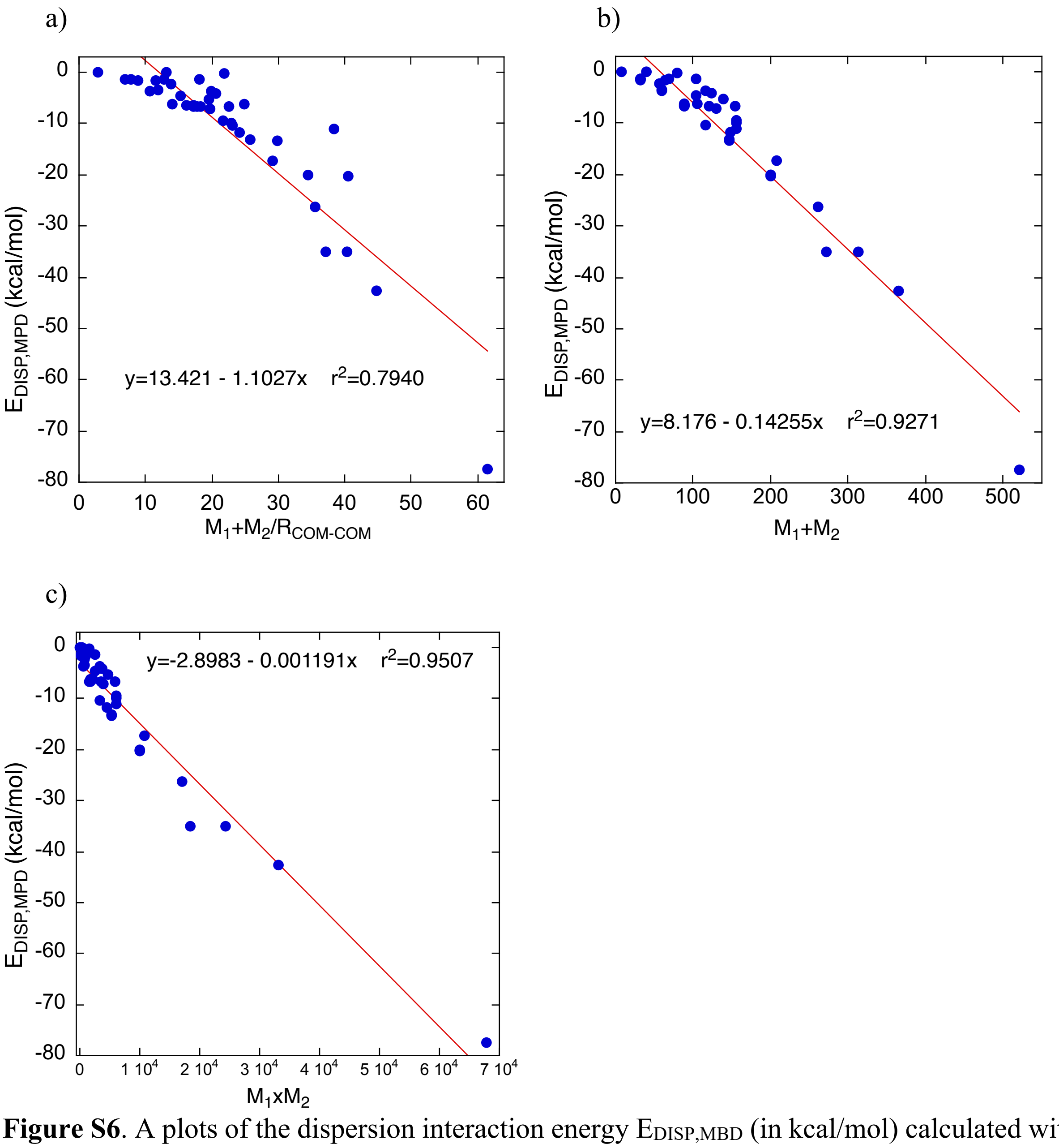

Figure S6. A plots of the dispersion interaction energy $E_{DISP,MBD}$ (in kcal/mol) calculated with MBD correction vs.: M_1+M_2/$R_{COM-COM}$ (a), M_1+M_2 (b), and M_1xM_2 (c) for the entire set of the dimers used in this study

Full citation for Ref. 29

Evgeny Epifanovsky, Andrew T. B. Gilbert, Xintian Feng, Joonho Lee, Yuezhi Mao, Narbe Mardirossian, Pavel Pokhilko, Alec F. White, Marc P. Coons, Adrian L. Dempwolff, Zhengting Gan, Diptarka Hait, Paul R. Horn, Leif D. Jacobson, Ilya Kaliman, Jörg Kussmann, AdrianW. Lange, Ka Un Lao, Daniel S. Levine, Jie Liu, Simon C. McKenzie, Adrian F. Morrison, Kaushik D. Nanda, Felix Plasser, Dirk R. Rehn, Marta L. Vidal, Zhi-QiangYou, Ying Zhu, Bushra Alam, Benjamin J. Albrecht, Abdulrahman Aldossary, Ethan Alguire, Josefine H. Andersen, Vishikh Athavale, Dennis Barton, Khadiza Begam, Andrew Behn, Nicole Bellonzi, Yves A. Bernard, Eric J. Berquist, Hugh G. A. Burton, Abel Carreras, Kevin Carter-Fenk, Romit Chakraborty, Alan D. Chien, Kristina D. Closser, Vale Cofer-Shabica, Saswata Dasgupta, Marc de Wergifosse, Jia Deng, Michael Diedenhofen, Hainam Do, Sebastian Ehlert, Po-Tung Fang, Shervin Fatehi, Qingguo Feng, Triet Friedhoff, James Gayvert, Qinghui Ge, Gergely Gidofalvi, Matthew Goldey, Joe Gomes, Cristina E. González-Espinoza, Sahil Gulania, Anastasia O. Gunina, Magnus W. D. Hanson-Heine, Phillip H. P. Harbach, Andreas Hauser, Michael F. Herbst, Mario Hernández Vera, Manuel Hodecker, Zachary C. Holden, Shannon Houck, Xunkun Huang, Kerwin Hui, Bang C. Huynh, Maxim Ivanov, Ádám Jász, Hyunjun Ji, Hanjie Jiang, Benjamin Kaduk, Sven Kähler, Kirill Khistyaev, Jaehoon Kim, Gergely Kis, Phil Klunzinger, Zsuzsanna Koczor-Benda, Joong Hoon Koh, Dimitri Kosenkov, Laura Koulias, Tim Kowalczyk, Caroline M. Krauter, Karl Kue, Alexander Kunitsa, Thomas Kus, István Ladjánszki, Arie Landau, Keith V. Lawler, Daniel Lefrancois, Susi Lehtola, Run R. Li, Yi-Pei Li, Jiashu Liang, Marcus Liebenthal, Hung-Hsuan Lin, You-Sheng Lin, Fenglai Liu, Kuan-Yu Liu, Matthias Loipersberger, Arne Luenser, Aaditya Manjanath, Prashant Manohar, Erum Mansoor, Sam F. Manzer, Shan-Ping Mao, Aleksandr V. Marenich, Thomas Markovich, Stephen Mason, Simon A. Maurer, Peter F. McLaughlin, Maximilian F. S. J. Menger, Jan-Michael Mewes, Stefanie A. Mewes, Pierpaolo Morgante, J. Wayne Mullinax, Katherine J. Oosterbaan, Garrette Paran, Alexander C. Paul, Suranjan K. Paul, Fabijan Pavoševi´c, Zheng Pei, Stefan Prager, Emil I. Proynov, Ádám Rák, Eloy Ramos-Cordoba, Bhaskar Rana, Alan E. Rask, Adam Rettig, Ryan M. Richard, Fazle Rob, Elliot Rossomme, Tarek Scheele, Maximilian Scheurer, Matthias Schneider, Nickolai Sergueev, Shaama M. Sharada, Wojciech Skomorowski, David W. Small, Christopher J. Stein, Yu-Chuan Su, Eric J. Sundstrom, Zhen Tao, Jonathan Thirman, Gábor J. Tornai, Takashi Tsuchimochi, Norm M. Tubman, Srimukh Prasad Veccham, Oleg Vydrov, Jan Wenzel, Jon Witte, Atsushi Yamada, Kun Yao, Sina Yeganeh, Shane R. Yost, Alexander Zech, Igor Ying Zhang, Xing Zhang, Yu Zhang, Dmitry Zuev, Alán Aspuru-Guzik, Alexis T. Bell, Nicholas A. Besley, Ksenia B. Bravaya, Bernard R. Brooks, David Casanova, Jeng-Da Chai, Sonia Coriani, Christopher J. Cramer, György Cserey, A. Eugene DePrince III, Robert A. DiStasio Jr., Andreas Dreuw, Barry D. Dunietz, Thomas R. Furlani, William A. Goddard III, Sharon Hammes-Schiffer, Teresa Head-Gordon, Warren J. Hehre, Chao- Ping Hsu, Thomas-C. Jagau, Yousung Jung, Andreas Klamt, Jing Kong, Daniel S. Lambrecht, WanZhen Liang, Nicholas J. Mayhall, C. William McCurdy, Jeffrey B. Neaton, Christian Ochsenfeld, John A. Parkhill, Roberto Peverati, Vitaly A. Rassolov, Yihan Shao, Lyudmila V. Slipchenko, Tim Stauch, Ryan P. Steele, Joseph E. Subotnik, Alex J. W. Thom, Alexandre Tkatchenko, Donald G. Truhlar, Troy Van Voorhis, Tomasz A. Wesolowski, K. Birgitta Whaley, H. Lee Woodcock III, Paul M. Zimmerman, Shirin Faraji, Peter M. W. Gill, Martin Head-Gordon, John M. Herbert, and Anna I. Krylov. Software for the frontiers of quantum chemistry: An overview of developments in the Q-Chem 5 package. *J. Chem. Phys*. **2021**, *155*, 084801.

Table S8. Bond lengths between the closest H atoms of the dimers (R_{H-H}, in Å), corresponding MBD and D4 dispersion interaction energies ($E_{DISP,MBD}$ and $E_{DISP,D4}$, in au)[a]

	R_{H-H}	$E_{DISP,MBD}$	$E_{DISP,D4}$
1	2.425	-0.00208376	-0.0017843
2	2.500	-0.00217864	-0.0020411
3	2.850	-0.00221213	-0.0024345
4	2.600	-0.002658071	-0.0030396
7	2.375	-0.005496169	-0.0050074
8	2.575	-0.00592966	-0.0068
9	2.450	-0.00670277	-0.0089262
10	2.330	-0.00994873	-0.00930195
11	2.530	-0.01051204	-0.01159069
12	2.425	-0.01071294	-0.01367970
13	2.225	-0.0102895	-0.00964333
14	2.125	-0.0164187	-0.01561769
15	2.450	-0.00726311	0.00753346
16	2.275	-0.0274201	-0.0292114
17	2.075	-0.0558462	-0.0560462
18	2.275	-0.0149274	-0.0190080
19	2.325	-0.0157359	-0.0168938
20	2.175	-0.04170417	-0.0435226
21	2.125	-0.05575758	-0.0579044
22	2.10	-0.067968895	-0.0708924
23	1.975	-0.12338109	-0.1293960
24	2.375	-0.0115002	-0.0121986
25	2.450	-0.00593488	-0.0054693
26	2.263	-0.01880351	-0.0193925
27	2.50	-0.00856597	-0.0111620
28	2.250	-0.01080644	-0.0101855

a) Calculated with cc-pVTZ basis set. b) Calculated with the following masses of the corresponding atoms: C=12.0107 amu, H=1.00794 amu, F=18.998 amu, Si=28.085 amu

Table S9. Squared bond lengths between the closest H atoms in the corresponding dimers (R^2_{H-H}, in Å), squares of distance between the respective centers of masses ($R^2_{com-com}$, in Å), the corresponding $E_{DISP,MBD}$ (kcal/mol) values.[a] Shown also the respective products of the monomer's masses[b] (M_1xM_2, in amu), M_1xM_2/R^2_{H-H} and M_1xM_2/$R^2_{com-com}$ values.

	R^2_{H-H}	$R^2_{com-com}$	$E_{DISP,MBD}$ (kcal/mol)	M_1xM_2 (amu)	M_1xM_2/R^2_{H-H}	M_1xM_2/$R^2_{com-com}$
1	5.8806	21.1830	-1.31	257.35731	43.76378	12.149235
2	6.2500	28.6236	-1.37	1158.2451	185.3192	40.46469
3	8.1225	32.8994	-1.39	2706.34883	333.1916	82.2613
4	6.760	31.0829	-1.67	1031.50169	152.5890	33.1855
5		16.4990	-1.46	257.35731		15.59654
6		13.2256	-1.65	257.35731		19.45903
7	5.6406	26.0488	-3.45	904.14717	160.29273	34.709778
8	6.6306	34.2564	-3.73	3398.77340	512.58912	99.21485
9	6.0025	36.3163	-4.21	3871.07952	644.91121	106.59344
10	5.4056	39.5779	-6.24	1944.43252	359.7071	49.12925

11	6.4009	45.6922	-6.60	3620.54924	565.63128	79.23780
12	5.8806	47.2491	-6.72	5981.55294	1017.16712	126.59612
13	4.9506	25.8949	-6.46	1944.43252	392.7670	75.08940
14	4.5156	25.4339	-10.30	3378.21338	748.1206	122.82326
15	6.0025	40.7772	-4.55	2711.84312	451.7856	66.50391
16	5.1756	50.7813	-17.21	10847.3724	2095.8676	213.60959
17	4.3056	53.6234	-35.04	18560.79264	4310.8493	346.13233
18	5.1756	52.0476	-9.37	6101.45330	1178.8881	117.22833
19	5.4056	46.3529	-9.87	6101.45330	1128.7282	131.63045
20	4.7306	54.4246	-26.17	17135.0718	3622.1773	314.84056
21	4.5156	59.8720	-34.99	24405.84444	5404.78440	407.63369
22	4.4100	66.4192	-42.65	33219.07212	7532.66942	500.14261
23	3.9006	71.7731	-77.42	67793.99498	17380.4017	944.560076
24	5.6406	43.2977	-7.22	4067.702188	721.147074	93.94730
25	6.0025	31.3398	-3.72	707.3994147	117.850798	22.571919
26	5.1212	37.8348	-11.80	4592.600987	896.782187	121.385629
27	6.250	50.6503	-5.38	4811.971229	769.915397	95.003805
28	5.0625	26.3621	-6.78	1747.684771	345.221683	66.2953547
29		23.0275	-6.71	1944.423703		84.4391216
30		29.4480	-6.36	1944.423703		66.029086
31		23.8037	-13.36	5329.91984		223.911729
32		32.2056	-13.11	5329.91984		165.49655
33		24.4441	-20.22	10040.42878		410.750185
34		33.6597	-20.13	10040.42878		298.292081
35		16.4787	-11.08	6101.459548		370.262766
36		16.5023	-2.33	786.979786		47.6891511
37		18.1093	-6.25	2191.283945		121.00337
38		9.31715	-0.06	407.2202921		43.7065495
39		13.4234	-0.18	1595.842704		118.884864
40		7.5076	-0.006	16.02080678		2.13394517

a) Calculated with cc-pVTZ basis set. b) Calculated with the following masses of the corresponding atoms: C=12.0107 amu, H=1.00794 amu, F=18.998 amu, Si=28.085 amu

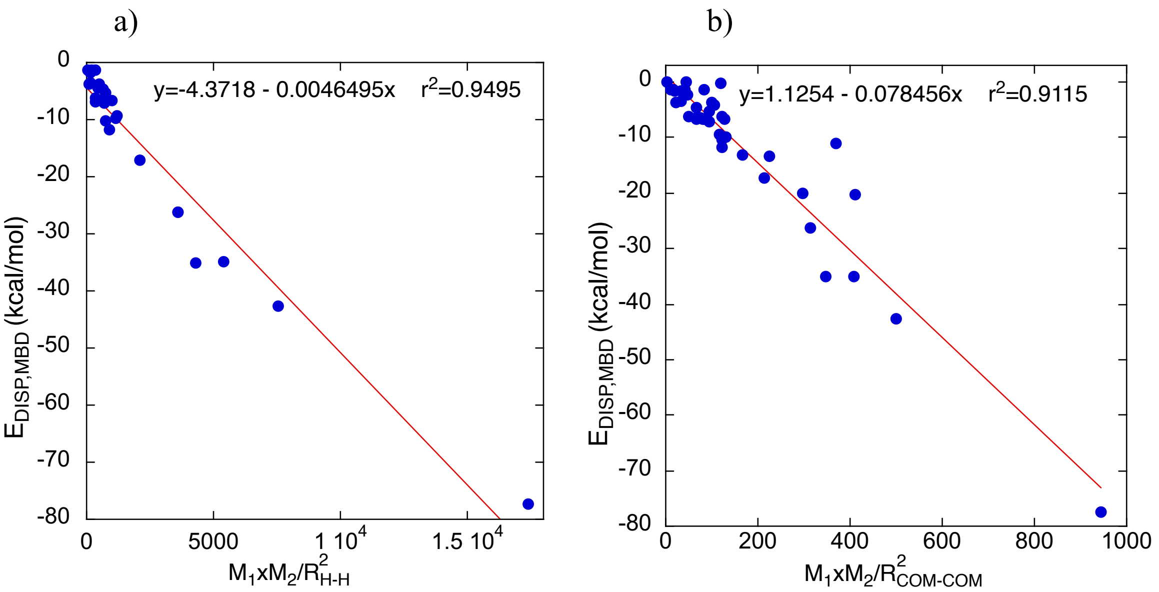

Figure S7. Dependence of the dispersion interaction energy (E_{DISP} (kcal/mol)) on M_1xM_2/R^2_{H-H} (a) for 26 dimers and on $M_1xM_2/R^2_{com-com}$ (b) for 40 dimers. M (in amu) and R (in Å).

Table S10. Bond lengths between closest H atoms of the dimers (R_{H-H}, in Å), MBD dispersion interaction energies of dimers ($E_{DISP,MBD}$, in kcal/mol), products of masses of monomers (M_1xM_2, in amu), and M_1xM_2/R_{H-H} values.

	R_{H-H}	$E_{DISP,MBD}$ (kcal/mol)	M_1xM_2 (amu)	M_1xM_2/R_{H-H}
1	2.425	-1.31	257.35731	113.5494
2	2.500	-1.37	1158.2451	463.29804
3	2.850	-1.39	2706.34883	949.59608
4	2.600	-1.67	1031.50169	396.73142
7	2.375	-3.45	904.14717	380.69355
8	2.575	-3.73	3398.77340	1319.9120
9	2.450	-4.21	3871.07952	1580.0325
10	2.330	-6.24	1944.43252	835.5204
11	2.530	-6.60	3620.54924	1431.0471
12	2.425	-6.72	5981.55294	2466.6198
13	2.225	-6.46	1944.43252	783.9023
14	2.125	-10.30	3378.21338	1589.7475
15	2.450	-4.55	2711.84312	1106.8747
16	2.275	-17.21	10847.3724	4768.0758
17	2.075	-35.04	18560.79264	8944.9603
18	2.275	-9.37	6101.45330	2681.9575
19	2.325	-9.87	6101.45330	2624.2810
20	2.175	-26.17	17135.0718	7878.1939
21	2.125	-34.99	24405.84444	11485.1033
22	2.10	-42.65	33219.07212	15818.6078
23	1.975	-77.42	67793.99498	34326.0734
24	2.375	-7.22	4067.702188	1712.71671
25	2.450	-3.72	707.3994147	288.73446

26	2.263	-11.80	4592.600987	2029.43040
27	2.50	-5.38	4811.971229	1924.78849
28	2.250	-6.78	1747.684771	776.748787

C=12.0107 amu, H=1.00794 amu, F=18.998 amu, Si=28.085 amu

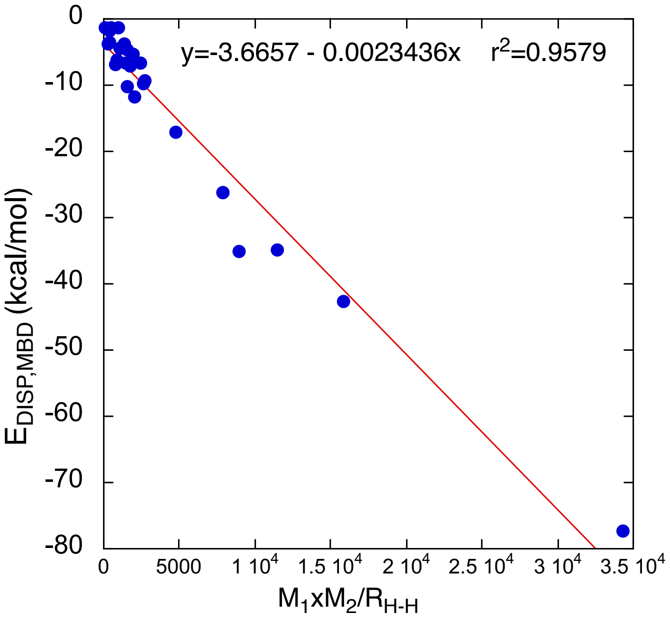

Figure S8. Dependence of MBD dispersion interaction energy (E_{DISP} (kcal/mol)) for 26 dimers, on M_1xM_2/R_{H-H} (M in amu and R_{H-H} in Å).

Cartesian Coordinates of investigated dimers in Å optimized by PBE0-MBD/cc-pVTZ

Dimer 1: $H_3CH—HCH_3$

	X	Y	Z
C	-0.00002328	-0.00001050	2.29962938
H	0.00004709	0.00009262	1.21074120
H	-0.44879776	0.92380972	2.66167945
H	-0.57566855	-0.85064087	2.66153549
H	1.02444564	-0.07325096	2.66160300
H	-0.00004833	0.00009262	-1.21438946
C	0.00002204	-0.00001049	-2.30327765
H	0.57566731	-0.85064087	-2.66518376
H	-1.02444688	-0.07325095	-2.66525127
H	0.44879652	0.92380973	-2.66532771

Dimer 2: $H_2FCH—HCFH_2$

C	-0.0064833782	0.0004280546	2.3825481057
H	-0.0028495232	0.0001943428	1.2903348389
H	-0.4526951536	0.9278654166	2.7470696809
H	-0.5800926742	-0.8541083059	2.7468470985

F	1.2861503661	−0.0919328182	2.8382998089
H	0.0026626832	0.0001943428	−1.2096585590
C	0.0062965382	0.0004280646	−2.3018718355
H	0.5799058342	−0.8541082959	−2.6661708185
H	0.4525083136	0.9278654266	−2.6663934009
F	−1.2863372061	−0.0919328082	−2.7576235389

Dimer 3: HF_2CH—HCF_2H

C	−0.0013978207	0.0047607409	2.7368992072
H	0.0107662146	−0.0212453544	1.6437564003
H	−0.4464506530	0.9222759180	3.1300146818
F	1.2670226819	−0.0957162406	3.1871654994
F	−0.7072707997	−1.0532899475	3.1879435224
H	−0.0104231088	0.0149023035	−1.2058642463
C	0.0056366944	−0.0093114802	−2.2989848953
H	0.5772991042	−0.8538258262	−2.6918572918
F	−1.2638710306	−0.0913303781	−2.7497339127
F	0.5532938478	1.1389474746	−2.7500296550

Dimer 4: H_3SiH—$HSiH_3$

Si	0.0000720282	0.0001118256	3.2722750414
H	0.0001559846	0.0001392535	1.7847462961
H	−0.6130750309	1.2620649212	3.7672411842
H	−0.7863461610	−1.1620321858	3.7670185323
H	1.3996784935	−0.1002678444	3.7671925426
H	−0.0000687746	0.0001392535	−0.8155949678
H	0.7864333524	−1.1620321770	−2.7978672130
H	−1.3995912836	−0.1002678343	−2.7980412225
H	0.6131622312	1.2620649408	−2.7980898542
Si	0.0000151803	0.0001118370	−2.3031237189

Dimer 5: H_2CH_2—H_2CH_2

C	−0.0000000000	2.0310878233	0.0000018800
H	0.0000000000	1.4014598716	0.8887371115
H	−0.0000000000	1.4014496727	−0.8887188101
H	0.8894424744	2.6597618570	−0.0000370907
H	−0.8894424744	2.6597618570	−0.0000370907
C	−0.0000000000	−2.0310878233	0.0000018800
H	0.0000000000	−1.4014496727	−0.8887188101
H	0.0000000000	−1.4014598716	0.8887371115
H	−0.8894424744	−2.6597618570	−0.0000370907
H	0.8894424744	−2.6597618570	−0.0000370907

Dimer 6: HCH_3—H_3CH

C	−0.0067554976	1.8190587381	0.0116687848
H	−0.0067311567	2.9078354823	0.0116712601
H	1.0194019665	1.4545440872	0.0116681266
H	−0.5198038312	1.4545358649	0.9003310037

H	−0.5198009982	1.4545357859	−0.8769885942
C	0.0067554957	−1.8181639614	−0.0116687871
H	0.0067311567	−2.9069405079	−0.0116712601
H	−1.0194020604	−1.4536500046	−0.0116681266
H	0.5198010417	−1.4536417023	0.8769886692
H	0.5198038735	−1.4536417822	−0.9003310864

Dimer 7: $H_3CH_2CH—HCH_2CH_3$

C	0.00214882	0.00000000	−0.00224742
H	−0.00466804	0.00000000	1.08975279
C	1.42012592	0.00000000	−0.54734243
H	−0.55323542	−0.88051111	−0.33175574
H	−0.55323542	0.88051111	−0.33175574
H	1.42751418	0.00000000	−1.63949900
H	1.97480398	0.88028972	−0.21577444
H	1.97480398	−0.88028972	−0.21577444
H	−0.00466804	0.00000000	3.43985059
C	0.00214882	0.00000000	4.53185080
C	1.42012592	0.00000000	5.07694581
H	−0.55323542	−0.88051111	4.86135912
H	−0.55323542	0.88051111	4.86135912
H	1.42751418	0.00000000	6.16910238
H	1.97480398	−0.88028972	4.74537782
H	1.97480398	0.88028972	4.74537782

Dimer 8: $H_3CH_2SiH—HSiH_2CH_3$

Si	−0.1010958100	0.0000000000	−0.3850109700
H	−0.2411142100	−0.0000000000	1.0978039001
C	1.7112119400	0.0000000000	−0.8724718600
H	−0.7942154600	−1.2085756800	−0.9158247300
H	−0.7942154600	1.2085756800	−0.9158247300
H	1.8243794100	−0.0000000000	−1.9579014200
H	2.2164259900	0.8836743700	−0.4782551000
H	2.2164259900	−0.8836743700	−0.4782551000
H	−0.2411142100	0.0000000000	3.6728038999
C	1.7112119500	−0.0000000000	5.6430796700
H	−0.7942154600	−1.2085756900	5.6864325300
H	−0.7942154600	1.2085756900	5.6864325300
H	1.8243794100	0.0000000000	6.7285092300
H	2.2164259900	−0.8836743700	5.2488629200
H	2.2164259900	0.8836743700	5.2488629200
Si	−0.1010958000	−0.0000000000	5.1556187900

Dimer 9: $H_3SiH_2SiH—HSiH_2SiH_3$

Si	−0.0985782884	−0.0000000000	−0.3850552762
H	−0.2324978226	−0.0000000000	1.0992089122
H	−0.7851083206	−1.2103069019	−0.9191453956
H	−0.7851083206	1.2103069019	−0.9191453956
Si	2.1668504884	−0.0000000000	−0.9931796202
H	2.3120735491	−0.0000000000	−2.4765197504

H	2.8500298123	1.2098479018	-0.4534419386
H	2.8500298123	-1.2098479018	-0.4534419386
H	-0.2324978126	0.0000000000	3.5492089278
Si	-0.0985782684	0.0000000000	5.0334731262
Si	2.1668504884	0.0000000000	5.6415974802
H	-0.7851083206	-1.2103069119	5.5675632556
H	-0.7851083206	1.2103069119	5.5675632556
H	2.3120735591	0.0000000000	7.1249376104
H	2.8500298223	-1.2098479018	5.1018597986
H	2.8500298223	1.2098479018	5.1018597986

Dimer 10: $C_2H_5H_2CH—HCH_2C_2H_5$

C	0.00223849	-0.00000004	0.07990961
H	-0.00158362	-0.00000006	1.17161102
C	1.41438867	0.00000019	-0.48207322
H	-0.55149327	-0.88112347	-0.25492001
H	-0.55149337	0.88112333	-0.25492002
H	1.95687221	-0.87438829	-0.10928119
H	1.95687156	0.87438930	-0.10928174
C	1.42801063	-0.00000008	-2.00148519
H	0.91383256	0.88093120	-2.39491708
H	0.91383259	-0.88093140	-2.39491702
H	2.44432752	-0.00000005	-2.40014561
H	-0.00158367	-0.00000106	3.50161104
C	0.00223838	-0.00000050	4.59331245
C	1.41438854	0.00013863	5.15529527
H	-0.55158039	0.88106889	4.92814104
H	-0.55140644	-0.88117793	4.92814306
H	1.95678569	0.87458042	4.78250289
H	1.95695771	-0.87419718	4.78250402
C	1.42801059	0.00013969	6.67470723
H	0.91391891	-0.88084195	7.06813939
H	0.91374620	0.88102064	7.06813886
H	2.44432750	0.00023929	7.07336759

Dimer 11: $C_2H_5H_2SiH—HSiH_2C_2H_5$

Si	-0.1005063408	0.0000000125	-0.3657123425
H	-0.2486606207	0.0000000118	1.1171749902
C	1.7095072207	-0.0000000340	-0.8857786218
H	-0.7882293453	-1.2069636920	-0.9113439784
H	-0.7882293515	1.2069637102	-0.9113439830
H	2.1970643357	0.8746627344	-0.4442021341
H	2.1970642093	-0.8746629008	-0.4442021938
C	1.8775556631	0.0000000198	-2.4025190994
H	1.4111488562	0.8795426842	-2.8524546635
H	2.9301480907	-0.0000006274	-2.6945909523
H	1.4111477220	-0.8795419484	-2.8524548791
H	-0.2486606140	0.0000000117	3.6534328148
C	1.7095072219	-0.0000000341	5.6563864343
H	-0.7882293433	-1.2069636922	5.6819517824
H	-0.7882293491	1.2069637096	5.6819517869
H	2.1970642137	-0.8746628999	5.2148100063

H	2.1970643387	0.8746627361	5.2148099487
Si	−0.1005063377	0.0000000129	5.1363201498
C	1.8775556575	0.0000000131	7.1731269111
H	2.9301480860	−0.0000006214	7.4651987694
H	1.4111488417	0.8795426784	7.6230624774
H	1.4111477241	−0.8795419540	7.6230626864

Dimer 12: $(H_3CSiH_2)H_2SiH$— $HSiH_2(SiH_2CH_3)$

Si	−0.0987678764	0.0000000203	−0.3114442820
H	−0.2402185968	0.0000000104	1.1725223719
H	−0.7944377060	−1.2077142583	−0.8445536237
H	−0.7944377057	1.2077142984	−0.8445536230
Si	2.1600444331	−0.0000000491	−0.9475293444
H	2.8211637522	1.2030639819	−0.3576795021
H	2.8211635719	−1.2030642234	−0.3576795922
C	2.3592051882	0.0000000200	−2.8210994994
H	1.8921622695	0.8834227915	−3.2599668958
H	3.4151114277	−0.0000005988	−3.0982056930
H	1.8921611822	−0.8834220727	−3.2599671056
H	−0.2402185869	0.0000000101	3.5980854280
H	−0.7944376966	−1.2077142586	5.6151614332
H	−0.7944377058	1.2077142983	5.6151614228
H	2.8211635719	−1.2030642234	5.1282874021
H	2.8211637522	1.2030639819	5.1282873114
Si	−0.0987678751	0.0000000206	5.0820520837
C	2.3592051712	0.0000000109	7.5917073096
H	3.4151114294	−0.0000005878	7.8688135174
H	1.8921622524	0.8834227801	8.0305747040
H	1.8921611930	−0.8834220739	8.0305749053
Si	2.1600444343	−0.0000000483	5.7181371519

Dimer 13: $(CH_3)_2HCH$—$HCH(CH_3)_2$

C	−0.00130921	0.00829586	0.06878500
H	0.01781991	−0.02488842	1.16258167
C	1.42054500	−0.00720493	−0.46623394
H	−0.52533523	−0.89800841	−0.24997121
H	1.42600366	−0.00356350	−1.55954882
H	1.97296456	0.87618300	−0.13476537
H	1.97258547	−0.88852289	−0.13349594
C	−0.77920788	1.23154588	−0.38527400
H	−0.84161167	1.27267983	−1.47589903
H	−1.79805269	1.23223575	0.00732171
H	−0.29169578	2.15093822	−0.05031088
H	0.01781991	−0.02488842	3.38846564
C	−0.00130921	0.00829586	4.48226227
C	1.42054501	−0.00720493	5.01728124
H	−0.52533523	−0.89800841	4.80101852
H	1.42600366	−0.00356350	6.11059611
H	1.97258547	−0.88852289	4.68454323

H	1.97296456	0.87618300	4.68581266
C	-0.77920788	1.23154589	4.93632131
H	-1.79805269	1.23223575	4.54372557
H	-0.84161167	1.27267982	6.02694631
H	-0.29169578	2.15093822	4.60135817

Dimer 14: $(CH_3)_3CH—HC(CH_3)_3$

C	-0.01867003	-0.00026251	0.00660356
H	0.02542256	-0.00099163	1.10205595
C	1.40986154	0.00025070	-0.51775215
H	1.95896592	0.88367179	-0.18271678
H	1.95966049	-0.88245404	-0.18198103
C	-0.76614511	1.25072724	-0.43186753
H	1.41881553	-0.00015332	-1.61215676
H	-0.83291619	1.29295460	-1.52333352
H	-1.78530201	1.26262972	-0.03794077
H	-0.26394951	2.16003879	-0.09389782
C	-0.76608832	-1.25083971	-0.43313634
H	-0.25281504	-2.16067104	-0.11371236
H	-1.77827828	-1.27330395	-0.02199639
H	-0.85140731	-1.28159662	-1.52367120
H	0.02542256	-0.00099163	3.22706809
C	-0.01867004	-0.00026251	4.32252049
C	1.40986154	0.00025070	4.84687620
H	1.41881553	-0.00015332	5.94128081
H	1.95966049	-0.88245404	4.51110508
H	1.95896592	0.88367179	4.51184084
C	-0.76614512	1.25072724	4.76099158
H	-1.78530202	1.26262972	4.36706483
H	-0.83291619	1.29295460	5.85245757
H	-0.26394951	2.16003879	4.42302186
C	-0.76608833	-1.25083971	4.76226038
H	-1.77827828	-1.27330395	4.35112044
H	-0.25281504	-2.16067105	4.44283642
H	-0.85140731	-1.28159662	5.85279525

Dimer 15: $H_3C_4H—HC_4H_3$

C	-0.0001687437	-0.0000420774	0.3614840648
C	-0.8121542346	-0.2455295356	-0.8377639905
C	0.1935361579	0.8263002974	-0.8374772692
C	0.6187193391	-0.5806060723	-0.8375169626
H	-0.0000279918	-0.0000907350	1.4299437628
H	-1.7773919736	-0.5377258467	-1.1932536006
H	0.4228979272	1.8082958658	-1.1930569814
H	1.3544594196	-1.2702281584	-1.1932987107
C	-0.0001783350	-0.0000353000	4.9484985106
C	-0.8121120231	-0.2455622293	6.1478344539
C	0.1935314708	0.8262186893	6.1475830681
C	0.6186901035	-0.5806212054	6.1475563725
H	-0.0000324003	-0.0000809641	3.8800014420
H	-1.7769127997	-0.5376922744	6.5044606346
H	0.4228204561	1.8077584147	6.5043676979

H	1.3541035677	−1.2699780186	6.5044196277

Dimer 16: $H_7C_8H—HC_8H_7$ (cubane)

C	−2.37438447	−0.01391040	0.09884370
H	−1.28799640	−0.03512861	0.15212364
C	−4.14778752	−0.93512832	−0.83041188
C	−4.12750489	1.22660964	−0.39560995
C	−3.20643343	0.25290551	−1.19176459
C	−4.23643748	−0.22901780	1.25693113
C	−3.31541388	−1.20282062	0.46084844
C	−3.29511100	0.95905424	0.89568028
C	−5.06903241	0.03775498	−0.03358762
H	−4.49182155	−1.69883200	−1.52468842
H	−4.45488733	2.20468479	−0.74189213
H	−2.79034514	0.44620851	−2.17829474
H	−4.65224637	−0.42236391	2.24356374
H	−2.98771769	−2.18079631	0.80710684
H	−2.95068094	1.72266937	1.58984614
H	−6.15558078	0.05880427	−0.08645048
C	2.05734830	0.00538088	−0.06151765
H	0.97096030	0.02659937	−0.11479860
C	2.97807526	−0.96758450	−0.85835285
C	2.99837839	1.19429062	−0.42352221
C	3.91940243	0.22048707	−1.21960352
C	2.88939603	−0.26143458	1.22909153
C	3.81046792	−1.23513946	0.43293829
C	3.83075080	0.92659878	0.86773902
C	4.75199612	−0.04628527	0.07091613
H	2.63364560	−1.73119990	−1.55251858
H	2.67068285	2.17226622	−0.76978149
H	4.33521233	0.41383252	−2.20623583
H	2.47330675	−0.45473693	2.21562140
H	4.13784970	−2.21321450	0.77922136
H	4.17478444	1.69030273	1.56201542
H	5.83854442	−0.06733485	0.12378001

Dimer 17: $H_{15}C_{10}H—HC_{10}H_{15}$ (adamantane)

C	−1.2512580945	0.0014447688	−4.5331335448
C	−0.0001235489	1.2445251646	−2.7612423124
C	1.2481335316	−0.0013367292	−4.5311413071
C	−0.0029283957	−1.2565268787	−2.7691903511
C	−1.2503012639	−1.2486921591	−3.6528854863
C	−0.0006594466	0.0020950040	−5.4138613643
C	−1.2467375929	1.2467239390	−3.6461299866
C	1.2473963684	1.2436834160	−3.6447126890
C	1.2453783869	−1.2511955542	−3.6514603233
C	−0.0026505360	−0.0089965776	−1.8844115499
H	−2.1450794262	0.0043889516	−5.1648857415
H	0.0009877220	2.1356012499	−2.1257000212
H	2.1418366957	−0.0004543894	−5.1618030462
H	−0.0038482437	−2.1525328115	−2.1403679585
H	2.1455059233	−1.2737565650	−3.0272449767

H	1.2692678550	−2.1503564250	−4.2769208835
H	−0.0009339824	−0.8777868157	−6.0657312229
H	−1.2754300701	−2.1478464803	−4.2782930880
H	−2.1504389072	−1.2698005946	−3.0287598493
H	−0.8848964964	−0.0102264960	−1.2357477040
H	0.0010895729	0.8848621166	−6.0618708738
H	0.8778642353	−0.0123636823	−1.2336945592
H	−2.1479497683	1.2694405004	−3.0236737797
H	1.2698733945	2.1461993692	−4.2654872765
H	−1.2663652760	2.1493731136	−4.2667886778
H	2.1487325387	1.2637698441	−3.0222209102
H	3.8360448211	0.0002950370	−6.3613914539
C	4.7298358432	0.0010844825	−6.9919263799
C	4.7303085767	1.2481697816	−7.8756345239
C	4.7330471126	−1.2464687062	−7.8748194344
C	5.9783164308	0.0029481456	−6.1088865644
C	5.9773801566	1.2523639250	−8.7598945071
H	4.7076765449	2.1490254113	−7.2526031839
H	3.8288296955	1.2686817563	−8.4978754295
C	5.9807806158	−1.2475248312	−8.7579867258
H	3.8320676507	−1.2689344965	−8.4978189406
H	4.7112191662	−2.1476227034	−7.2521085138
C	7.2287109083	0.0062218143	−6.9896685254
H	5.9796434423	−0.8795548254	−5.4604383280
H	5.9753753912	0.8830178962	−5.4572372534
C	7.2245631611	1.2521204877	−7.8754596256
C	5.9782475715	0.0016408752	−9.6407077164
H	5.9761534374	2.1461192284	−9.3917461569
C	7.2279643357	−1.2408795855	−7.8736092341
H	5.9837006737	−2.1426289557	−9.3878126810
H	8.1226008273	0.0075858975	−6.3578267144
H	7.2461857452	2.1548254344	−7.2550714782
H	8.1256650763	1.2722286312	−8.4982189724
H	6.8586688519	0.0016709201	−10.2918095343
H	5.0963929708	0.0001414723	−10.2900312493
H	8.1290236854	−1.2603983485	−8.4964381158
H	7.2516713723	−2.1413794735	−7.2504037938

Dimer 18: $H_5C_6H—HC_6H_5$ (benzene)

C	−1.8886433495	0.4272969555	−0.0023207220
C	−0.5011088215	0.4270111546	−0.0019362631
C	0.1907353463	1.6294635249	−0.0000457465
C	−0.5009853069	2.8319913063	0.0012476671
C	−1.8885294418	2.8318375519	0.0007452587
C	−2.5816137257	1.6295992353	−0.0008973882
H	−2.4307227463	−0.5111284562	−0.0036850862
H	0.0424161171	−0.5105849175	−0.0029922318
H	0.0426887290	3.7695039862	0.0027632426
H	−2.4305864714	3.7702796370	0.0016667416
H	−3.6653376103	1.6296568270	−0.0009757388
H	1.2740659167	1.6293969842	0.0005073145
C	4.6329088738	1.6293763963	0.0024390280
C	5.3249574838	1.6277637110	1.2049445003

C	5.3260483855	1.6310847575	-1.1994379419
C	6.7125496307	1.6280234319	1.2052165362
H	4.7825718668	1.6263473713	2.1431521121
C	6.7136497778	1.6314609720	-1.1984511490
H	4.7844567544	1.6321542240	-2.1381079604
C	7.4066060820	1.6299793310	0.0037030499
H	7.2541525322	1.6268150955	2.1438195148
H	7.2561739899	1.6330264855	-2.1365247806
H	8.4904328331	1.6304335782	0.0042129079
H	3.5493067642	1.6293964868	0.0019374947

Dimer 19: $H_5C_6H—HC_6H_5$ (Triprismane)

C	-0.6654161080	-0.0678866446	-0.6372746920
C	0.8274273122	-0.2367315119	-0.4705247402
C	0.2164639032	1.1460808224	-0.4520997173
C	-0.8495023996	-0.1686820622	0.8961582965
C	0.6437916028	-0.3384413441	1.0630290165
C	0.0336278928	1.0445883386	1.0815210985
H	-1.2945932140	-0.3394860683	-1.4727392445
H	-1.6586624173	-0.5294803097	1.5149923483
H	0.0331422162	1.7804870751	1.8726843724
H	1.1974607327	-0.8510236290	1.8366211687
H	1.5468765138	-0.6637314526	-1.1545050480
H	0.3820330911	1.9816718787	-1.1167034110
C	0.9048528427	4.3647830623	-3.4932383800
C	-0.0096443177	4.9397555519	-4.6007343307
C	2.1194049337	4.6480382267	-4.3481484240
C	1.6120534554	5.6959922099	-3.3836851428
C	1.2043848752	5.2238486867	-5.4558717023
C	0.6964596074	6.2721319328	-4.4913447243
H	-1.0235854992	4.7218008138	-4.9040702742
H	3.0767964731	4.1474500448	-4.3716046268
H	2.1103967585	6.1413844085	-2.5346799130
H	1.2887048858	5.2610177827	-6.5324903683
H	0.3221584948	7.2663977248	-4.6892264553
H	0.7533846244	3.6043423929	-2.7407535763

Dimer 20: $H_9C_{10}H—HC_{10}H_9$ (Pentaprismane)

C	-0.2524600070	-1.0133455480	-1.3084510286
C	1.2023428909	-0.6801342326	-0.8600567007
C	-0.5691593379	0.4981829316	-1.4079848244
C	-0.5902209567	-1.4817671976	0.1277872283
C	0.8855714273	0.8327998953	-0.9591324579
C	0.8650055577	-1.1465025881	0.5770294549
C	-1.1044470424	0.9647410555	-0.0329415372
C	-1.1153066994	-0.2577621954	0.9165880389
C	0.3502350756	1.2997032063	0.4164666313
C	0.3394462547	0.0777885855	1.3649913630
H	2.0858098912	-1.0751905679	-1.3590849713
H	-1.0182674282	0.8909791720	-2.3188994937
H	-1.0510233414	-2.4565990155	0.2788110131
H	1.5501816685	1.4840261726	-1.5244825314

H	1.5169913655	−1.8630801646	1.0739856731
H	−1.9229990685	1.6813872172	0.0087838480
H	−1.9403844277	−0.3861589833	1.6154650559
H	0.6444926639	2.2741908901	0.8031900561
H	0.6268124576	0.2066334536	2.4072871760
H	−0.4825668974	−1.6637823038	−2.1502485480
C	−0.7138634707	−3.6457930115	−4.7080799530
C	−0.4123438040	−5.1606791128	−4.6110405153
C	−2.1763598896	−3.9634756864	−5.1428221942
C	−0.3840570508	−3.1815950449	−6.1474921840
C	0.1043515776	−5.6336930377	−5.9909146209
C	−1.8748256562	−5.4796841804	−5.0459485470
H	0.0415899904	−5.5578881175	−3.7044500564
C	−1.8471215428	−3.5008517957	−6.5830218950
H	−3.0510613421	−3.5589723149	−4.6359986414
C	0.1199106049	−4.4116312329	−6.9407528893
H	0.0861544968	−2.2120280105	−6.3032607761
C	−1.3581995382	−5.9525854164	−6.4264608017
H	0.9140524241	−6.3598334943	−6.0404490003
H	−2.5406188269	−6.1235645651	−4.4736099424
C	−1.3425653281	−4.7309797595	−7.3753750645
H	−2.4955613129	−2.7769212742	−7.0739068558
H	0.9399080320	−4.2924099727	−7.6471930399
H	−1.6673627213	−6.9235425482	−6.8104016185
H	−1.6411534792	−4.8567877106	−8.4148874217
H	−0.4692830292	−2.9978037567	−3.8684757682

Dimer 21: $H_{11}C_{12}H$—$HC_{12}H_{11}$ (Hexaprismane)

C	−0.7937701194	−3.9693770211	−4.7237895521
C	−0.6010539242	−5.4878778222	−4.4913639555
C	−2.2499761921	−4.2270055090	−5.2044260931
C	−0.4498223636	−3.3471050274	−6.0980853218
C	−0.0688192361	−6.3865351320	−5.6335518465
C	−2.0570530735	−5.7437206207	−4.9711506386
H	−0.2696374056	−5.7639816393	−3.4906853364
C	−1.9048459527	−3.6054998308	−6.5796552724
H	−3.1066620159	−3.7840830727	−4.6971486332
C	0.0881995877	−4.2442937497	−7.2381553574
H	−0.0325673881	−2.3412261262	−6.0611849776
C	−1.5250201606	−6.6397890709	−6.1143385878
H	0.5770115408	−7.2051661188	−5.3168566836
C	−1.3668415239	−4.5002908053	−7.7221949313
H	−2.5568001372	−2.7917982883	−6.8963273398
H	0.8330989979	−3.7777212461	−7.8821057995
H	−1.9484419014	−7.6430751463	−6.1541549920
H	−1.6922728153	−4.2206467014	−8.7238037985
H	−0.5815145091	−3.3386504448	−3.8610192820
C	0.2782824250	−5.7632082978	−7.0064975535
H	1.1347696239	−6.2051612447	−7.5148520329
C	−1.1777193850	−6.0181950699	−7.4891464687
H	−1.3903695664	−6.6509038666	−8.3503729471
H	−2.7966199842	−6.2109703017	−4.3222520155
C	−4.7519484066	−8.0855193054	−2.5924408034

C	-6.2494919294	-8.0312141936	-2.9784575731
C	-4.3396970362	-8.2291919293	-1.1086764830
C	-4.7702648079	-9.6328107867	-2.7392581034
C	-7.3374004249	-8.1223022573	-1.8821157238
H	-6.4884497605	-7.3747216644	-3.8146342906
C	-6.2680281364	-9.5785743602	-3.1254882109
C	-5.4273147360	-8.3148008218	-0.0120716823
H	-3.4335174395	-7.6948698773	-0.8243117927
C	-4.3608570410	-9.7773028164	-1.2530350686
H	-4.1241845804	-10.1476624001	-3.4498411845
H	-8.2302213413	-7.5245508528	-2.0637074126
C	-6.9266052039	-8.2636965621	-0.3970432869
C	-7.3530610559	-9.6703887121	-2.0266242606
H	-6.5219820182	-10.0588313534	-4.0700831469
H	-5.1752428432	-7.8279346821	0.9295761923
C	-5.4463255806	-9.8625944844	-0.1535866944
H	-3.4714742490	-10.3810241737	-1.0744956688
H	-7.5706454788	-7.7481208855	0.3147702193
C	-6.9432692394	-9.8118061864	-0.5404053375
H	-8.2557073997	-10.2106841095	-2.3102979890
H	-5.2041329076	-10.5127242776	0.6865900763
H	-7.6013248211	-10.4344451985	0.0650080132
H	-4.0957875939	-7.4632659956	-3.1994031012

Dimer 22: $H_{13}C_{14}H$—$HC_{14}H_{13}$ (Heptaprismane)

C	-2.0835613695	-0.3726119953	-0.1371003795
C	-1.4077653649	-1.7457682381	0.1059386653
C	-0.9002861813	-1.7566870137	-1.3655586290
C	-1.5754432043	-0.3838157126	-1.6082164625
C	-0.9812568972	1.0335036253	-1.4148389338
C	-1.4897114651	1.0452658743	0.0561074028
C	-0.0732149561	1.4418294461	0.5421361136
C	0.4349431715	1.4302246025	-0.9287403129
C	1.6073704464	0.5068191416	-0.5159445722
C	1.0994390826	0.5173448886	0.9551996415
C	1.1451801075	-1.0310477971	0.9821428459
C	0.0300933917	-2.0378543347	0.6035047554
C	0.5369878492	-2.0478576667	-0.8674823983
C	1.6512666959	-1.0407528267	-0.4891049419
H	-3.1552724148	-0.4005144531	0.0644883383
H	-2.1202599805	-2.5020935665	0.4381881449
H	-2.2945594193	-0.4188337069	-2.4278421716
H	-2.2465209985	1.7695796484	0.3605496571
H	-1.2600467990	-2.5204228289	-2.0564770215
H	0.0812514328	-2.9500512781	1.1997459066
H	0.9414650801	-2.9676204995	-1.2927650484
H	-1.3849244047	1.7500697246	-2.1315245179
H	0.7829535085	2.3565824628	-1.3878134286
H	-0.0774641579	2.3755500461	1.1062625903
H	1.7163835762	0.9606941361	1.7380310702
H	2.5786757269	0.9415468265	-0.7567907911
H	1.7882631644	-1.4101941881	1.7776177574
H	2.6456737745	-1.4264147855	-0.7157975748

C	5.5855584725	−2.6093754144	−0.9559979780
C	6.5150517092	−1.7252800391	−1.8355339199
C	6.4913339271	−2.3439585809	0.2726703423
C	5.6762598376	−3.7488536444	−2.0011793596
C	7.4232104233	−1.4609727578	−0.6077474181
C	6.6078778692	−2.8649663334	−2.8806382828
H	6.1307436415	−0.8006753747	−2.2688310130
C	7.7163208303	−3.1561421749	0.7566184850
H	5.9402120931	−1.8934954161	1.0994588706
C	6.7015497686	−4.9072518845	−2.0764302503
H	4.6946430230	−4.0406577545	−2.3772772417
C	8.6488816913	−2.2744700222	−0.1235012909
H	7.5240201381	−0.3965318636	−0.3915019538
C	7.6336320840	−4.0235217483	−2.9550435422
H	6.2755267945	−2.5456655575	−3.8693552509
C	8.3363854713	−4.4327697985	0.1342528317
H	7.8203873960	−3.1368844986	1.8423749146
C	7.8867511347	−5.2120768883	−1.1261605174
H	6.2653505997	−5.8154846297	−2.4946455502
C	9.2701343362	−3.5508058095	−0.7448292681
H	9.4001049021	−1.6412649844	0.3508559542
C	8.8197344092	−4.3285829662	−2.0053045569
H	7.8452241820	−4.3192524826	−3.9835764256
H	8.7686828490	−5.0892727846	0.8907074934
H	8.0790409466	−6.2822584217	−1.0384614833
H	10.3502900401	−3.5947449135	−0.5984509192
H	9.6596224477	−4.7847707818	−2.5310698952
H	4.5556240371	−2.2981388454	−0.7786724199

Dimer 23: $H_{39}C_{40}H$—$HC_{39}H_{40}$ Dodecahedrane

C	0.4417432342	2.9412859596	1.9010751563
C	−0.1758873784	1.7585296238	1.1268205557
H	0.6972751322	2.6480236008	2.9204699273
H	−0.2318262815	0.8690771531	1.7562468602
C	0.4481016628	2.9414415545	−2.1406292836
C	1.7004702543	3.4107281317	−1.3667322833
H	0.7064595403	2.6467768704	−3.1591458628
H	2.5910651037	3.3547821819	−1.9942447021
C	−0.5721867518	4.0979843685	−2.1428077965
C	−1.8269181850	3.6282494889	−1.3723758885
H	−0.8275461121	4.3909985926	−3.1623266628
H	−2.7156032692	3.6837704629	−2.0026447722
C	−0.5783842799	4.0981349875	1.8993432659
C	0.0414831666	5.2805909554	1.1264961870
H	−0.8370528202	4.3914429244	2.9179509736
H	0.0954194559	6.1704883088	1.7555072150
C	−1.5797983466	2.1812926842	0.6481958716
H	−2.3436131265	1.5062892506	1.0373799482
C	1.4468072734	4.8576879160	0.6523526790
H	2.2095764820	5.5325226269	1.0439036393
C	1.4492811464	4.8576270177	−0.8895004929
H	2.2132690880	5.5323761804	−1.2788032836
C	−1.5772839758	2.1816617536	−0.8935878214

H	−2.3390718647	1.5058181847	−1.2858872339
C	0.6928353278	1.4984962712	−0.1190475963
H	1.0734829921	0.4756581393	−0.1194210807
C	−1.9802487724	4.5229060754	−0.1238894928
H	−2.9463668904	5.0301535779	−0.1254504760
C	−0.8234216957	5.5406654980	−0.1221783949
H	−1.2052575777	6.5628433538	−0.1228671703
C	1.8500239565	2.5158466540	−0.1175641713
H	2.8159590199	2.0082174890	−0.1160327120
C	0.0453838518	5.2804966920	−1.3681140525
H	0.1013385974	6.1702321918	−1.9971732889
C	−1.8306202829	3.6282998062	1.1252923179
H	−2.7213127695	3.6841462901	1.7526938741
C	1.6963605437	3.4108065782	1.1307047085
H	2.5850646136	3.3551373104	1.7609384030
C	−0.1717254817	1.7610515233	−1.3663459047
H	−0.2256673410	0.8724454764	−1.9952411216
H	−0.3310909127	−0.7320938128	−3.1446243720
C	−0.3843524056	−1.6204466530	−3.7739349789
C	1.0218973106	−2.0405784855	−4.2450449942
C	−1.0050502168	−2.8013362615	−3.0012049390
C	−1.2474018668	−1.3572890848	−5.0221393351
C	1.0266583665	−2.0390269732	−5.7867783070
H	1.7831145146	−1.3649864711	−3.8512501680
C	1.2711046199	−3.4873929505	−3.7667829419
C	0.0153993473	−3.9577551561	−2.9983077318
C	−2.2561980299	−3.2704880606	−3.7772304267
H	−1.2649191852	−2.5071653054	−1.9829133787
C	−2.4040692931	−2.3750754524	−5.0261664334
H	−1.6291265008	−0.3348523610	−5.0210783931
C	−0.3766769893	−1.6159042577	−6.2670433875
H	1.7912325079	−1.3639318887	−6.1743259900
C	1.2783741251	−3.4857342298	−6.2643871405
H	2.1589126873	−3.5431012430	−3.1353106848
C	1.4263452223	−4.3812032648	−5.0155382302
H	0.2693951585	−4.2514039083	−1.9786214877
C	−0.6009309683	−5.1399424868	−3.7746627866
H	−3.1478009602	−3.2147429920	−3.1511456215
C	−2.0041788622	−4.7170306533	−4.2550488685
C	−2.2483609901	−3.2689076401	−6.2747811456
H	−3.3703631069	−1.8682144535	−5.0290130756
H	−0.3201967579	−0.7256027511	−6.8952832742
C	−0.9928229742	−2.7984768784	−7.0431139744
C	0.0274349320	−3.9552530571	−7.0405441706
H	2.1700131583	−3.5410619879	−6.8904808204
C	0.2697103699	−5.3991409607	−5.0195685516
H	2.3925093424	−4.8883470299	−5.0129454526
H	−0.6576404914	−6.0300477427	−3.1461852711
C	−1.9993637208	−4.7160246380	−5.7969786321
H	−2.7686859223	−5.3921287067	−3.8673621023
H	−3.1361510662	−3.2127985220	−6.9062273807
H	−1.2468498094	−2.5047661631	−8.0627776687
C	−0.5933108098	−5.1383208749	−6.2693732440
H	0.2877142646	−4.2479217550	−8.0589330928

H	0.6516042704	-6.4212973592	-5.0189494101
H	-2.7614562272	-5.3906708720	-6.1901501884
H	-0.6461201203	-6.0277974813	-6.8990749443

Dimer 24: H_5C_6H—Tetrahedrane

C	-0.8072131836	-0.6872439668	-0.0175338828
C	0.6857423740	-0.8560504161	0.1492237906
C	0.0746863487	0.5266863632	0.1677370793
C	-0.9912073284	-0.7879780934	1.5159520303
C	0.5021124699	-0.9576940409	1.6828293924
C	-0.1081025396	0.4254050124	1.7014067276
H	-1.4365452604	-0.9584934451	-0.8528879327
H	-1.8001423106	-1.1489328333	2.1348878138
H	-0.1088468230	1.1614143186	2.4923418496
H	1.0555756835	-1.4705756230	2.4563037488
H	1.4054303432	-1.2826590582	-0.5346262675
H	0.2404765757	1.3621437705	-0.4967879826
H	0.6196666538	3.0199175874	-2.1558853145
C	0.7905369323	3.7655312361	-2.9024151626
C	0.2867244234	5.0174513742	-3.4831912598
C	0.9231751791	4.0120810542	-4.3449978250
C	1.7339298609	4.7810097445	-3.3904244205
H	-0.4816374694	5.7585692288	-3.4274979323
H	0.9097871940	3.5582120498	-5.3130521433
H	2.6845433467	5.2415655273	-3.2247161485

Dimer 25: $(C_2H_5)H_2CH$—HCH_3

H	-0.0014146100	-0.0000008500	4.3674171100
C	0.0023330100	-0.0000004900	5.4591480200
C	1.4145922200	0.0001383100	6.0207931600
H	-0.5512135600	0.8812307100	5.7941256100
H	-0.5510399700	-0.8813396100	5.7941271800
H	1.9567109800	0.8747777800	5.6480102800
H	1.9568826300	-0.8743954600	5.6480113300
C	1.4278454600	0.0001393500	7.5401739300
H	0.9135484000	-0.8810027900	7.9331436400
H	0.9133757200	0.8811807700	7.9331431800
H	2.4441837700	0.0002389500	7.9388489900
H	-0.0014172700	-0.0000011100	1.9174171100
C	-0.0013997500	-0.0000100600	0.8285159400
H	0.5114311600	-0.8898722000	0.4664784900
H	-1.0285213800	0.0007658100	0.4666085500
H	0.5127174300	0.8891103900	0.4664891500

Dimer 26: Cubane—$HCH(CH_3)_2$

H	0.0180062346	-0.0250547974	4.2202116197
C	-0.0012311017	0.0083822258	5.3139799327
C	1.4206623521	-0.0099303285	5.8488348373
H	-0.5267913423	-0.8970670867	5.6327671492
H	1.4263059151	-0.0063962179	6.9421740166

H	1.9706669102	−0.8924359983	5.5159121322
H	1.9748898269	0.8722531386	5.5172364839
C	−0.7769197955	1.2331793298	5.7678709540
H	−1.7957548037	1.2359510225	5.3752742388
H	−0.8393479404	1.2745523370	6.8585131410
H	−0.2876560440	2.1516298876	5.4329280667
H	0.0177656222	−0.0248711365	1.9569652626
C	0.0179178890	−0.0249300120	0.8693837721
C	0.6246380448	1.0936032948	−0.0300430693
C	0.6829261337	−1.1105319095	−0.0292142422
C	−1.2547437666	−0.0589002345	−0.0301471796
C	1.2902292367	0.0081428631	−0.9297081011
C	−0.6473692812	1.0597276626	−0.9306695869
H	1.1140869061	1.9953403916	0.3326751528
C	−0.5890890673	−1.1443209867	−0.9298234093
H	1.2202690658	−1.9845227020	0.3335586032
H	−2.2799295019	−0.0867734392	0.3332184735
C	0.0176862515	−0.0258333350	−1.8298406915
H	2.3155160165	0.0360067684	−1.2926753646
H	−1.1847904336	1.9337987027	−1.2930361230
H	−1.0786462397	−2.0461460057	−1.2920674013
H	0.0182030028	−0.0259911449	−2.9176074575

Dimer 27: $H_3CSiH_2H_2SiH$—$HSiH_2SiH_2$

H	−0.2394799800	0.0000000100	4.5482453700
H	−0.7949319600	−1.2077581000	6.5646492900
H	−0.7949319700	1.2077581400	6.5646492800
H	2.8213041300	−1.2034116500	6.0783499600
H	2.8213043100	1.2034114000	6.0783498700
Si	−0.0986036500	0.0000000200	6.0325704600
C	2.3591351000	0.0000000200	8.5407023200
H	3.4150547500	−0.0000005900	8.8173577000
H	1.8920650300	0.8835308000	8.9793157500
H	1.8920639800	−0.8835301000	8.9793159500
Si	2.1604396700	−0.0000000500	6.6676036700
H	−0.2408528600	0.0000000100	2.0482457500
Si	−0.2402637900	0.0000000100	0.5586805800
Si	−2.4417266300	0.0000000100	−0.2510077600
H	0.4906756400	−1.2102360900	0.0871784800
H	0.4906756400	1.2102361100	0.0871784800
H	−2.4524933700	0.0000000100	−1.7412748200
H	−3.1700562600	−1.2097774000	0.2258242400
H	−3.1700562600	1.2097774200	0.2258242400

Dimer 28: $(CH_3)_3CH$—HCH_2CH_3

H	−0.0260784541	0.0528243955	−1.9751729028
C	−0.0066980864	0.0135565051	−3.0709281182
C	−0.6117576848	1.3083727860	−3.5945521382
H	−0.6167373567	1.3176307096	−4.6889077278
H	−1.6438809062	1.4305448100	−3.2574056869

H	−0.0429132402	2.1786911305	−3.2584133704
C	1.4446248078	−0.1277754502	−3.5079506009
H	1.8930042496	−1.0423458684	−3.1128031511
H	1.5159835073	−0.1689705592	−4.5992038601
H	2.0475480653	0.7170237439	−3.1672440093
C	−0.8275433867	−1.1908336565	−3.5095761542
H	−0.4249283425	−2.1194850324	−3.0981219188
H	−1.8675017424	−1.1032721162	−3.1864516296
H	−0.8248955278	−1.2832636543	−4.6000545243
H	−0.0261054782	0.0528522113	0.2750683529
C	−0.0260686451	0.0528011412	1.3670157320
C	−0.9964133248	1.0820305166	1.9209025662
H	0.9970030801	0.2513738969	1.6930894390
H	−0.2843813126	−0.9567899893	1.6931062542
H	−0.9968579007	1.0824726235	3.0129551604
H	−0.7378575443	2.0909907623	1.5927655007
H	−2.0188817067	0.8831670841	1.5927905171

Dimer 29: H_8C_3-C_3H_8 (4C-12)

C	2.1911341907	3.3783829701	0.7001526766
C	3.1814456860	2.3215862728	0.2388471677
C	4.2852796373	2.8882099863	−0.6377432979
H	1.4046302831	2.9478043569	1.3236600037
H	4.9874909381	2.1139807287	−0.9539806140
H	1.7091428823	3.8654597634	−0.1516992904
H	2.6889186095	4.1567287460	1.2844570580
H	3.6241528325	1.8325568228	1.1126945110
H	2.6480692239	1.5367897146	−0.3072659481
H	3.8735406572	3.3510753982	−1.5386742567
H	4.8544097052	3.6561662207	−0.1069085733
C	2.1087665742	−1.8545554067	0.7506722393
C	3.3594612602	−1.9985778198	−0.1007788451
C	4.6377334198	−1.8731410540	0.7116510406
H	1.1994082942	−1.9498303783	0.1536611413
H	5.5266993394	−1.9781532739	0.0862149960
H	2.0816636388	−0.8801482543	1.2455626306
H	2.0759225197	−2.6192387062	1.5313336349
H	3.3444835582	−2.9666508891	−0.6110225378
H	3.3534207432	−1.2409531905	−0.8910441495
H	4.6929673838	−0.9005899898	1.2078752025
H	4.6849786228	−2.6405320180	1.4888652105

Dimer 30: H_8C_3-C_3H_8 (4C-13)

C	3.0492093650	−1.3117795113	0.6462699177
C	4.3374863270	−0.9043024106	−0.0494982514
C	5.5588430233	−1.0302508734	0.8458685432
H	2.1870628778	−1.2278559420	−0.0190984244
H	6.4729205975	−0.7392971917	0.3234507428
H	2.8555690043	−0.6835484255	1.5204239009
H	3.1003440348	−2.3473751616	0.9938900204
H	4.4781017381	−1.5190236868	−0.9445242163
H	4.2501365805	0.1274741797	−0.4047787900

H	5.4656882246	−0.3951846832	1.7309422072
H	5.6878686576	−2.0591181480	1.1931861098
C	0.1933149198	3.4403407928	0.7816593493
C	1.0324764881	3.4368005637	−0.4846901310
C	2.5137490863	3.2377726951	−0.2107540326
H	0.2920435198	2.4951118335	1.3228207819
H	3.0979463023	3.2491023493	−1.1334721555
H	−0.8666525365	3.5886285327	0.5633724949
H	0.5075254439	4.2392595863	1.4592025049
H	0.6771067889	2.6487957991	−1.1562298760
H	0.8853346450	4.3787938991	−1.0225442093
H	2.9034269502	4.0263645172	0.4388348993
H	2.6975379618	2.2818412858	0.2872986143

Dimer 31: $H_{12}C_5$-C_5H_{12} (6C-12)

C	2.3220386418	3.1285274296	0.7623409119
C	3.3063182934	2.1865516973	0.0903396719
C	4.5432756891	2.8932986861	−0.4412894784
C	5.5345489468	1.9594285917	−1.1157487726
C	6.7663922160	2.6742066729	−1.6450356624
H	1.4514173597	2.5939952863	1.1469248077
H	7.4655312145	1.9781629571	−2.1130312444
H	1.9622255010	3.8884375988	0.0638788113
H	2.7894635283	3.6502212698	1.6014261673
H	3.6132069194	1.4106324486	0.8007990473
H	2.8100786025	1.6603323980	−0.7327888024
H	4.2379895638	3.6713020223	−1.1520976955
H	5.0433390470	3.4191478801	0.3812114672
H	5.8406673060	1.1843330172	−0.4038889314
H	5.0318616958	1.4332281904	−1.9347425813
H	6.4979936858	3.4254287804	−2.3921414894
H	7.2990881588	3.1875851000	−0.8405338473
C	2.0210105999	−1.9646472909	0.9778711252
C	3.2067939208	−2.1838293264	0.0536416807
C	4.5411445837	−2.1680045665	0.7827018861
C	5.7356972100	−2.3874850644	−0.1315117739
C	7.0639906239	−2.3552610286	0.6057189963
H	1.0762851847	−1.9674291816	0.4300489415
H	7.9057688553	−2.5109930628	−0.0720909731
H	2.1006000749	−1.0057760958	1.4972522187
H	1.9658857222	−2.7466269373	1.7400907488
H	3.0945361483	−3.1395222719	−0.4706379534
H	3.2145243620	−1.4123835876	−0.7245295697
H	4.6563894940	−1.2101613650	1.3052894668
H	4.5368017472	−2.9365464837	1.5656142850
H	5.6226969037	−3.3480634878	−0.6462544259
H	5.7317074230	−1.6232564426	−0.9168524498
H	7.2139336768	−1.3940703184	1.1048882305
H	7.1078270998	−3.1325435153	1.3731271869

Dimer 32: $H_{12}C_5$-C_5H_{12} (6C-13)

C	3.0094003828	−1.6176852226	0.5015986250

C	4.2531277717	−1.4678970548	−0.3584478981
C	5.5444175355	−1.6072101478	0.4325472309
C	6.7978186700	−1.4589746692	−0.4152967678
C	8.0800874675	−1.5986665906	0.3883270241
H	2.0961101575	−1.5089346892	−0.0867990324
H	8.9653584168	−1.4943203078	−0.2422722928
H	2.9860161779	−0.8642037068	1.2940114287
H	2.9794060127	−2.5988681744	0.9830502612
H	4.2404001081	−2.2152326341	−1.1593874852
H	4.2403872801	−0.4924570180	−0.8568161819
H	5.5595866068	−0.8602463355	1.2357534654
H	5.5586909685	−2.5835886427	0.9321846643
H	6.7805781345	−2.2068618246	−1.2156417075
H	6.7818048824	−0.4835878392	−0.9138261968
H	8.1378023953	−0.8369799006	1.1699234137
H	8.1339419897	−2.5748931082	0.8770361272
C	0.0090058497	3.6429731369	0.7724058059
C	1.1226526949	3.3988645236	−0.2315476923
C	2.4839593875	3.2115716127	0.4190404206
C	3.6061228676	2.9696939585	−0.5771700993
C	4.9651210371	2.7887922128	0.0784840449
H	−0.0836167845	2.8059381944	1.4696508878
H	5.7497268063	2.6217067268	−0.6631288110
H	−0.9571567259	3.7718192668	0.2801319807
H	0.2020508997	4.5415910130	1.3644727210
H	0.8878167536	2.5141910412	−0.8340135409
H	1.1728254067	4.2368689878	−0.9357123794
H	2.7204486025	4.0954260623	1.0243780585
H	2.4356234798	2.3712859581	1.1226164754
H	3.3685226668	2.0848913045	−1.1788958552
H	3.6486582741	3.8082287583	−1.2815127595
H	5.2432444023	3.6714606917	0.6607052070
H	4.9656194241	1.9328644167	0.7591108579

Dimer 33: $H_{16}C_7$-C_7H_{16} (8C-12)

C	2.4010450741	−3.3889270290	−0.6145019342
C	3.3085997098	−2.2621250001	−0.1487446889
C	4.6444268168	−2.7566928323	0.3843296109
C	5.5654565923	−1.6400392317	0.8496684697
C	6.9048941298	−2.1336704088	1.3733452773
C	7.8274933504	−1.0133025148	1.8286661894
C	9.1661926521	−1.5130222693	2.3458941329
H	1.4491422323	−3.0109563721	−0.9928005657
H	2.1834592724	−4.0798701397	0.2041693816
H	2.8708810005	−3.9664292916	−1.4148946940
H	3.4879864482	−1.5655502290	−0.9750642540
H	2.8024174993	−1.6812038953	0.6298894064
H	4.4670763589	−3.4518156907	1.2136055865
H	5.1486803706	−3.3411656495	−0.3945587644
H	5.7367783070	−0.9424926396	0.0209013739
H	5.0629826322	−1.0587489569	1.6322257924
H	6.7369055507	−2.8266695491	2.2066311728
H	7.4062063671	−2.7185335782	0.5926431544

H	7.9896846684	−0.3221361102	0.9939427130
H	7.3267223802	−0.4299897673	2.6095778807
H	9.0326214796	−2.1831546527	3.1992229686
H	9.7045566339	−2.0688028999	1.5737051723
H	9.8063691485	−0.6889347367	2.6678684030
C	2.0721237461	2.1019856273	−0.6697057785
C	3.2264064964	2.3478888183	0.2870294286
C	4.5557245299	2.5516345784	−0.4234858421
C	5.7181252784	2.8034593193	0.5227730311
C	7.0533443504	2.9724734604	−0.1845589678
C	8.2124031564	3.2366393898	0.7633108690
C	9.5495879276	3.3612329692	0.0525122427
H	10.3631078893	3.5533328727	0.7551723074
H	1.1287884137	1.9618735454	−0.1375750927
H	2.2475783764	1.2087710807	−1.2752856595
H	1.9447343501	2.9435561182	−1.3562314352
H	3.0112749991	3.2255504003	0.9066281358
H	3.3165222735	1.5044091787	0.9805428374
H	4.7727697407	1.6702171585	−1.0392685060
H	4.4671966975	3.3913551986	−1.1234921003
H	5.5099209435	3.6972079426	1.1232614590
H	5.7908190616	1.9729574633	1.2352739567
H	7.2665644379	2.0722514030	−0.7741482651
H	6.9827750297	3.7934899034	−0.9084180398
H	8.0105836660	4.1509317102	1.3322848220
H	8.2622967520	2.4285778269	1.5016660939
H	9.7919846658	2.4449188880	−0.4924457949
H	9.5377885432	4.1795185917	−0.6725614862

Dimer 34: $H_{16}C_7$-C_7H_{16} (8C-13)

C	2.9945407732	−1.8076596393	0.2941995974
C	4.2566137206	−1.6917797434	−0.5438955216
C	5.5300312333	−1.8578100016	0.2712168593
C	6.8011614645	−1.7409879826	−0.5546041125
C	8.0731154846	−1.9041610892	0.2622702873
C	9.3453141512	−1.7726606976	−0.5605103762
C	10.6089324360	−1.9309618114	0.2686795644
H	2.0942234347	−1.6843282668	−0.3113717809
H	2.9346065830	−2.7835507904	0.7827909692
H	2.9735118853	−1.0469178934	1.0791633835
H	4.2408232311	−2.4423967610	−1.3417276785
H	4.2750388067	−0.7185458541	−1.0470198191
H	5.5461710345	−1.1082901713	1.0721193750
H	5.5117217117	−2.8323432237	0.7740584305
H	6.7852888455	−2.4912129856	−1.3543892658
H	6.8176546806	−0.7665822794	−1.0573774005
H	8.0851854086	−1.1595618928	1.0674957026
H	8.0633329786	−2.8820053568	0.7588558712
H	9.3337396833	−2.5190021334	−1.3625216444
H	9.3526909957	−0.7963301653	−1.0575309702
H	10.6593517300	−1.1726601752	1.0542607131
H	10.6410797841	−2.9094951679	0.7548717060
H	11.5081161159	−1.8350600770	−0.3433483644

C	0.0118411519	3.7639768075	1.1191401357
C	1.1419359688	3.5342622754	0.1300301782
C	2.4809615831	3.2789704522	0.8037982952
C	3.6214359887	3.0541442238	−0.1754104911
C	4.9638860935	2.8168016830	0.4971184318
C	6.1045465831	2.6020243869	−0.4846054124
C	7.4456108143	2.3768979277	0.1932350562
H	−0.1195029803	2.8990387779	1.7747383256
H	8.2444900890	2.2283271113	−0.5364390661
H	−0.9386536306	3.9427907258	0.6123130573
H	0.2160874459	4.6288963467	1.7559682287
H	0.8975122234	2.6853266081	−0.5179580182
H	1.2331931688	4.4019031038	−0.5326696364
H	2.7237836566	4.1272991783	1.4552946675
H	2.3916457869	2.4088240155	1.4656361209
H	3.3831578624	2.1991942829	−0.8199206981
H	3.7009702599	3.9204566693	−0.8434378465
H	5.2007480620	3.6695773444	1.1446820636
H	4.8885522325	1.9475483061	1.1618534427
H	5.8700128682	1.7460926077	−1.1273114116
H	6.1724962386	3.4683238379	−1.1520386935
H	7.7212079518	3.2315279682	0.8165490431
H	7.4188344084	1.4950995187	0.8387487020

Dimer 35: C_6H_6 --- C_6H_6

C	0.9812382036	−0.9812382036	−2.0303796619
C	−0.3588383700	−1.3396540419	−2.0299138414
C	1.3396540420	0.3588383700	−2.0299138414
C	−0.9812382036	0.9812382036	−2.0303796619
C	0.3588383700	1.3396540419	−2.0299138414
C	−1.3396540420	−0.3588383700	−2.0299138414
H	1.7475365316	−1.7475365315	−2.0254816961
H	−0.6400106814	−2.3863549279	−2.0252268750
H	−2.3863549280	−0.6400106813	−2.0252268751
H	−1.7475365316	1.7475365315	−2.0254816961
H	0.6400106814	2.3863549279	−2.0252268750
H	2.3863549280	0.6400106813	−2.0252268751
C	0.9812382036	−0.9812382036	2.0303796619
C	1.3396540420	0.3588383700	2.0299138414
C	0.3588383700	1.3396540419	2.0299138414
C	−0.9812382036	0.9812382036	2.0303796619
C	−1.3396540420	−0.3588383700	2.0299138414
C	−0.3588383700	−1.3396540419	2.0299138414
H	1.7475365316	−1.7475365315	2.0254816961
H	2.3863549280	0.6400106813	2.0252268751
H	0.6400106814	2.3863549279	2.0252268750
H	−1.7475365316	1.7475365315	2.0254816961
H	−2.3863549280	−0.6400106813	2.0252268751
H	−0.6400106814	−2.3863549279	2.0252268750

Dimer 36: C_2H_4 --- C_2H_4

C	−0.95963957	1.02733278	−2.03182198

C	0.31756923	1.36897317	-2.03180662
H	-1.75154858	1.76782901	-2.02709693
H	0.63398232	2.40603395	-2.02705839
H	1.10224852	0.62103065	-2.02709496
H	-1.26612262	-0.01255989	-2.02712693
C	0.31756923	1.36897317	2.03180662
C	-0.95963957	1.02733278	2.03182198
H	0.63398232	2.40603395	2.02705839
H	-1.75154858	1.76782901	2.02709693
H	1.10224852	0.62103065	2.02709496
H	-1.26612262	-0.01255989	2.02712693

Dimer 37: C_6H6 --- C_2H_4

C	0.9813883576	-0.9812832878	-2.0300376096
C	-0.3590331703	-1.3400783908	-2.0277835804
C	1.3396637768	0.3588974812	-2.0318365592
C	-0.9812832879	0.9813883576	-2.0300376096
C	0.3588974812	1.3396637768	-2.0318365576
C	-1.3400783909	-0.3590331703	-2.0277835820
H	1.7477621946	-1.7475407223	-2.0276763882
H	-0.6402951080	-2.3867533060	-2.0262953476
H	-2.3867533060	-0.6402951080	-2.0262953531
H	-1.7475407223	1.7477621945	-2.0276763882
H	0.6403299295	2.3861520785	-2.0272885231
H	2.3861520786	0.6403299294	-2.0272885287
C	1.3411122499	0.4060557297	2.0327668660
C	0.4060557296	1.3411122499	2.0327668636
H	2.3976105314	0.6501094434	2.0277093040
H	0.6501094434	2.3976105313	2.0277092985
H	1.0839142736	-0.6468807308	2.0296805842
H	-0.6468807309	1.0839142736	2.0296805808

Dimer 38: Ne -- Ne

Ne	0.0000000000	0.0000000000	-1.5262500000
Ne	0.0000000000	0.0000000000	1.5262500000

Dimer 39: Ar -- Ar

Ar	0.0000000000	0.0000000000	-1.8319000000
Ar	0.0000000000	0.0000000000	1.8219000000

Dimer 39: He -- He

He	0.0000000000	0.0000000000	-1.3700000000
He	0.0000000000	0.0000000000	1.3700000000
